# Short single-stranded DNA degradation products augment the activation of Toll-like receptor 9

**DOI:** 10.1038/ncomms15363

**Published:** 2017-05-22

**Authors:** Jelka Pohar, Duško Lainšček, Karolina Ivičak-Kocjan, Miša-Mojca Cajnko, Roman Jerala, Mojca Benčina

**Affiliations:** 1 Department of Synthetic Biology and Immunology, National Institute of Chemistry, Hajdrihova 19, SI-1001 Ljubljana, Slovenia; 2 EN-FIST Centre of Excellence, Trg Osvobodilne fronte 13, SI-1000 Ljubljana, Slovenia

## Abstract

Toll-like receptors encounter a diversity of degradation products in endosomes. TLR7 and TLR8 have been shown to be activated by RNA degradation products. Here we show that although TLR9 requires single-stranded DNA longer than 20 nucleotides for a robust response, TLR9 activation is augmented by CpG-containing oligodeoxyribonucleotides (sODNs) as short as 2 nucleotides, which, by themselves, do not induce activation in cell cultures, as well as in mice. sODNs also activate human TLR9 in combination with ODNs containing a single CpG motif that by themselves do not activate human TLR9. The specific sequence motif of sODN and colocalization of ODN and sODN suggest that the mechanism of activation involves binding of both ODN and sODN to TLR9. sODNs augment TLR9 activation by mammalian genomic DNA indicating the role of short DNA degradation products in the endosomes in response to infection or in autoimmune disease, particularly at limiting concentrations of ODNs.

## Abstract (web summary)

DNA degradation products are frequently found in the endosome, but how they regulate the activation of Toll-like receptors is not known. Here the authors show that single-stranded DNA as short as two nucleotides can enhance the ability of longer DNA oligonucleotides to activate Toll-like receptors.

Toll-like receptors (TLRs), as pattern recognition receptors, are critical for the recognition of the prototypical molecules characteristic of microbes, as well as autologous danger signals. The subgroup of TLRs (TLR3, TLR7, TLR8, TLR9 and TLR13) that reside in the endosomal compartments bind nucleic acids^1^. Synthetic oligodeoxyribonucleotides (ODN) with non-methylated-deoxycytidylyl-deoxyguanosine dinucleotide (CpG) motifs recapitulate receptor activation by natural substrates. Several types of CpG-containing ODNs, with distinct sequences and secondary structures, are potent activators of TLR9, inducing B-cell-proliferation, plasmacytoid dendritic cell (pDC) maturation, and the secretion of interleukin (IL)-6, IL-10, IL-12 and type I interferons^2^,^3^. Twelve nucleotides (nt) long B-class ODN and TLR9 were crystalized in a symmetric TLR9-CpG complex with a 2:2 stoichiometry^4^, identifying the residues involved in single-stranded DNA (ssDNA) recognition, which were distributed over several LRR segments of the TLR9 ectodomain. Minimal sequence motifs for B-class ODNs have been defined previously for human and mouse TLR9. The minimal B-class ODNs that activate human (h)TLR9 comprise two CpG motifs separated by 6–10 nt, with the first CpG motif being proceeded by the 5′-thymidine^5^. By contrast, mouse (m)TLR9 is activated by ODNs comprising a single CpG positioned 4–6 nt from the 5′-end^6^. A-class ODNs, which comprise oligomerizing segments, convert to B-class ODNs upon degradation via endosomal deoxyribonuclease (DNase) II^7^.

Nucleic acids are a common constituent of all microbes^8^, yet their activation of TLRs is contained within acidic cellular compartments with proteolytic and nuclease activity, which restricts activation by the circulating endogenous nucleic acids. In addition to the compartmentalization, limited activation by the autologous nucleic acids^9^ is accomplished by their selectivity against methylated-CpG motifs, the requirement for the proteolytic cleavage of the TLR9 ectodomain^10^, and by the acidic pH of the activating compartments^11^,^12^.

However, even in the absence of microbes, the destruction of host cells, including necrotic cell death and neutrophil extracellular traps (NETs), causes the release of nucleic acids, which can trigger sterile inflammation mediated by TLR9 (ref. 13). Extracellular unprotected cell-free (cf)DNA and NETs^14^ are processed by extracellular waste-management DNases before endocytosis^15^. Another sources of cfDNA are injuries and mitochondrial DNA, which is released during systemic inflammatory response syndrome^16^,^17^. On the other hand, confined microbial nucleic acids and autologous DNA associated with proteins-like histones, HMGB1, and autoantibodies^15^ are protected against the action of nucleases. DNA and its fragments are endocytosed such that they can encounter TLR9 in the endosomes before the DNA is completely degraded. DNase II is an endosomal acid-activated DNase that is necessary for the processing of genomic (g)DNA, and TLR9 activation by bacterial DNA is DNase-II-dependent^7^. The degradation of DNA generates a pool of DNA fragments, in which only ssDNA with unmethylated CpG motifs of appropriate lengths^5^,^6^ activate TLR9 and induce pDC activation, B-cell maturation and cytokine synthesis. Thus far, it has been assumed that small DNA fragments shorter than 10 nt have no role in the activation of TLR immune response. Even crystalized 12-nt ODN is a poor TLR9 activator^6^. However, it has been demonstrated that the small degradation products of RNA, such as uridine and uridine-guanosine dinucleotide, have a role in TLR8 activation^18^,^19^ and guanosine in TLR7 activation^20^.

Therefore, we investigated the potential role of short ssDNAs, as degradation products, in TLR9 activation. We found that short, CpG-containing ODNs (sODNs), while incapable of activating TLR9 by themselves, can augment the activation of TLR9 via CpG-containing stimulatory ODNs regardless of the phosphodiester (PD) or phosphothioate (PTO) backbone of the ODNs. This effect was observed in human and mouse cell lines, as well as *in vivo*. Also, sODNs promote the activation of hTLR9 by single CpG-containing ODNs, which, by themselves, do not activate hTLR9. Moreover, sODNs support the activation of TLR9 by mammalian gDNA, a poor activator of TLR9. Those results in combination with the previous reports on activation of TLR7 and TLR8 explain how the augmented activation of single-stranded nucleic acid sensing TLRs by short degradation products occurs in the degradation compartments, such as endosomes.

## Results

### sODNs comprising a CpG motif potentiate hTLR9 activation

**Figure 1:**
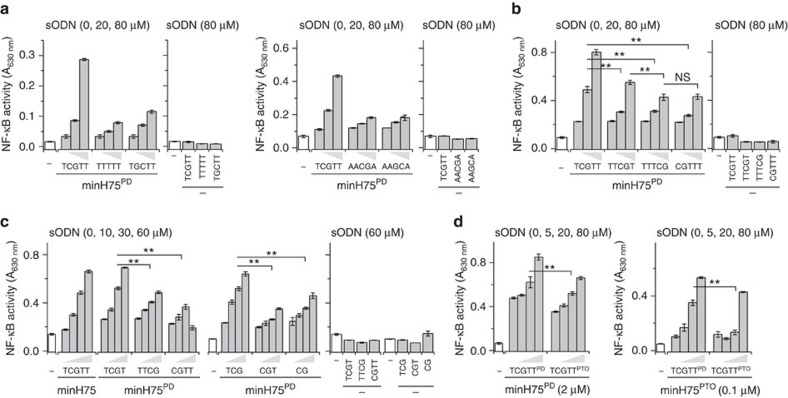
sODN combined with ODN enhances the activation of hTLR9. (**a**) CpG motif within sODNs is required to enhance the activation of hTLR9. (**b**) TCG motif at the 5^′^ of sODN is preferential for powered hTLR9 response. Ramos Blue cells were stimulated with minH75^PD^ (2 μM) and sODNs^PD^ (0, 20 and 80 μM) or with sODNs^PD^ (80 μM) alone as controls. See also Supplementary Fig. 1a. (**c**) CpG dinucleotide is sufficient to increase hTLR9 activation with minH ODN. Ramos Blue cells were stimulated with minH75^PD^ (2 μM) and sODNs^PD^ (0, 10, 30 and 60 μM) or with sODNs^PD^ (60 μM) alone as controls. (**d**) PD-based TCGTT augments the activation of hTLR9 more efficiently than PTO-based TCGTT. Ramos Blue cells were stimulated with a combination of minH75^PD^ (2 μM) or minH75^PTO^ (0.1 μM) and TCGTT^PD^ or TCGTT^PTO^ (0, 5, 20 and 80 μM). See also Supplementary Fig. 1b,c. (**a**–**d**) The NF-κB/AP-1-regulated expression of SEAP was analyzed 18 h after stimulation. (Data are representative of three independent experiments. Bars represent the means of five biological replicates±s.d.; **P<0.05, NS, *P*>0.05, unpaired two-tailed Student *t*-test).

Short ODNs in endosomes arise from the degradation of large double-stranded (ds)DNA. Therefore, heterogeneous DNA degradation products are likely present in endosomes. While it has been established that DNA fragments shorter than 10 nt are not able to activate TLR9 (refs 5, 6), they may, however, modulate TLR9 activation by longer ssODNs. Therefore, we exposed TLR9-expressing cells to a mixture of sODNs, in combination with established TLR9-stimulatory ODNs. For the initial experiments, we used Ramos Blue cells—a human B-lymphocyte cell line, which expresses secreted embryonic alkaline phosphatase (SEAP) under the control of an NF-κB/AP-1 promoter. No activation of hTLR9 was detected when the sODNs were administered alone (Fig. 1a); however, their addition to the stimulatory ODNs led to more than threefold increase in activation (Fig. 1a). This effect depended on the presence of a CpG motif within the sODNs. The sODNs with sequences other than TCGTT, when co-administered with minH75, exhibited a very small effect on cell activation (Fig. 1a and Supplementary Fig. 1a). Since the position of the CpG motif is important for hTLR9 activation^5^,^6^, we further examined whether the position of the CpG motif within the sODNs had any effect on the potency of the TLR9 augmentation. Five-nt sODNs with a CpG motif positioned from 5′- to 3′-end were tested. The CpG motif positioned adjacent to the 5′ T within the minH75 augmented TLR9 activation most effectively, followed by the TTCGT (Fig. 1b). The other sODNs, TTTCG and CGTTT, still augmented TLR9 activity but were significantly less efficient than TCGTT. In summary, sODNs comprising the CpG motif enhance hTLR9 activation when administered with TLR9-stimulatory minH75. Moreover, TCGTT, which shares the 5′-motif of ODNs that are potent TLR9 stimulators, most effectively augments the activation of TLR9 by minH75.

### CpG dinucleotide already enhances the activation of hTLR9

The initial sODN selection was the 5-nt ssDNA with a TCGTT sequence, which effectively enhanced the activation of hTLR9 in combination with minH75. We further investigated whether sODNs shorter than 5 nt improve TLR9 activation and whether the observed augmentation is driven by the position of the CpG motif (Fig. 1c). sODNs with a 5′TCG motif enhanced TLR9 activation by minH75 more effectively than sODNs with a CpG dinucleotide positioned at the 5′ end or 2 nt from the 5′ end (Fig. 1c). Surprisingly, the CpG dinucleotide also augmented the activity of TLR9 in a mixture with minH75; however, it was less effective compared to three-, four- and five-nucleotide long sODNs. sODNs administered alone had no effect on the activation of TLR9 (Fig. 1c). Also, sODNs longer than 5 nt with a 5′TCG motif augmented TLR9 response in the presence of minH75; however, sODNs longer than 7 nt also induced some activation of hTLR9 when added alone (Supplementary Fig. 1b,c). Therefore, for further experiments, TCGTT, which effectively augmented hTLR9 activation with minH75, was used.

Synthetic agonists based on the PD backbone may be degraded by DNAses generating sODNs that affect TLR9 activation, as was demonstrated for A-class ODN (ref. 7). Therefore, it is unclear whether the added sODNs are indeed the molecules that directly affect TLR9. To clarify this point, we assessed the effect of the PD and PTO backbone on the strength of TLR9 response augmentation (Fig. 1d). PTO- and PD-based ODNs have different TLR9 activation potencies^5^. Regardless of the backbone, TCGTT most potently augmented the activation of TLR9. However, interestingly, TLR9 response was significantly higher when the PD-backbone-based sODN was used, which indicates that the further degradation of sODNs is not likely to play a role, because shorter sODNs had weaker potency. Similarly, PD-based TCGTT, compared to PTO-based sODN, effectively enhanced TLR9 activation with PTO-based ODN (minH75; Fig. 1d). In combination with stimulatory ODN, CpG dinucleotide is sufficient to augment hTLR9 activity. A synergistic effect was seen for PD- and PTO-based TCGTT and minH75, which indicates that the sODN and ODN do not require further nuclease processing to affect TLR9 activation.

### CpG-sODN augments the activation of mTLR9

The above results highlight the synergistic effect of TCGTT and human-specific agonist minH75 on the activation of hTLR9. Next, we were interested whether sODNs improve the activation of mTLR9 in combination with mouse-specific ODNs because human and mouse TLR9 differ in terms of the sequence specificity of the CpG motifs within the stimulatory ODNs. To provide an equivalent cell framework with which to compare the effect of sODN on hTLR9 and mTLR9, we used HEK293 cells transfected with either hTLR9 or mTLR9. The cells were stimulated with human- or mouse-specific ODNs in combination with TCGTT. Regardless of the origin of TLR9 and the type of ODN, TCGTT augmented the TLR9 response (Supplementary Fig. 2). sODN promoted the activation of h and mTLR9 in the presence of C-type ODN2395, A-type ODNs (mODN1585, hODN2216; Supplementary Fig. 2a), B-type ODNs (minM80, minH75 and ODN1668; Supplementary Fig. 2b), and DNase II degradation products of hODN2216, A3_11 nt, A3_12 nt, and A3_13 nt (ref. 7; Supplementary Fig. 2c,d).

**Figure 2:**
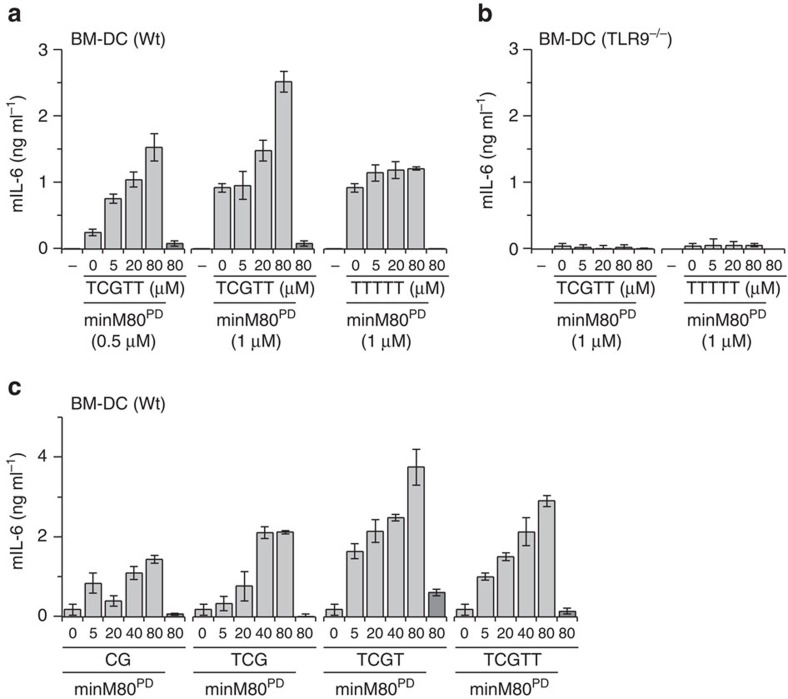
sODNs administered with agonistic ODNs augment the activation of mTLR9. (**a**,**b**) The effect of sODN with a CpG motif is TLR9-dependent. BM-DCs from wt mice (**a**) and TLR9^−/−^ mice (**b**) were stimulated with minM80^PD^ (1 μM), in combination with TCGTT^PD^ or TTTTT^PD^ (0-80 μM). (**c**) sODNs shorter than 5 nt augment BM-DC activation by minM80. The cells were stimulated with sODN^PD^s (0-80 μM) and minM80^PD^ (0.5 μM). (**a**–**c**) The synthesis of mIL-6 was analyzed 18 h later. (Data are representative of two independent experiments. Bars represent the means of four biological replicates ±s.d.). See also Supplementary Fig. 2 for activation of mTLR9-expressing HEK293.

To verify the results by more immunologically relevant cells, we used bone marrow-derived dendritic cells (BM-DCs) from wild-type (wt) and TLR9^−/−^ mice. TCGTT, but not TTTTT, augmented the activation of BM-DCs (Fig. 2a). This observed effect was exclusively linked to TLR9 because the TLR9^−/−^ BM-DCs did not respond to the ODN or sODN (Fig. 2b). Not only TCGTT but also CG, TCG and TCGT, in combination with minM80, augmented the secretion of mIL-6 among BM-DCs, with the most potent effect being found for the 4-nt TCGT (Fig. 2c).

### Short ssDNA is internalized into the endosomes

**Figure 3:**
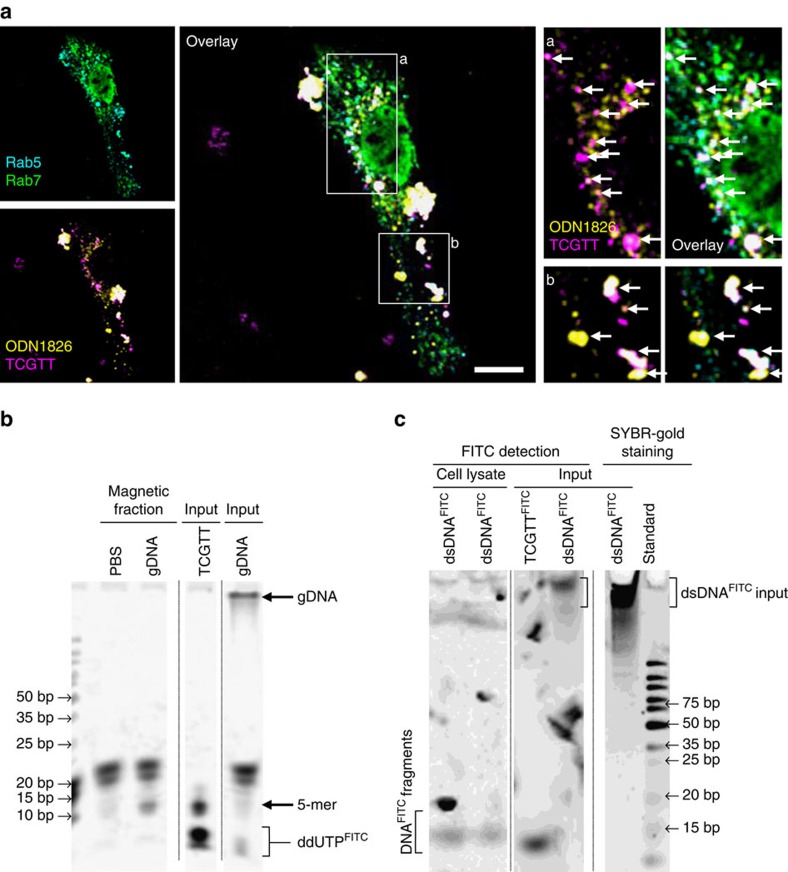
sODNs colocalize with agonistic ODN in endosomes. (**a**) ODN and sODN colocalize within endosomes. The BM-DCs from wt mice were treated with 5 μM TCGTT^PD-3′Cy3^ and 2 μM mouse-specific ODN1826^PD-3′Cy5^. 1 h later, the cells were fixed, and the spatial distribution of ODN and sODN was visualized in cells stained for Rab5 and Rab7 with anti-Rab5^ATTO390^ and anti-Rab7^AlexaFluor488^ antibodies. Arrows mark colocalization of ODN1826 and TCGTT with Rab5 or Rab7. See also Supplementary Fig. 3 for colocalization of hTLR9, ODN10104 and TCGTT. (Data are representative of three independent experiments). Scale bar, 10 μm. (**b**) Degraded mammalian gDNA (labeled with FITC after isolation from the endo/lysosomes) was detected in the enriched endo/lysosomal fraction. HEK293 cells were loaded with magnetic beads alone or together with calf thymus gDNA. After 16 h, the endo/lysosomes were enriched, and the fragmentation of the DNA was determined. The size marker is a GeneRuler Ultra-Low-Range DNA ladder (ThermoFisher Scientific). Input gDNA (5 μg) and TCGTT alone were loaded for size and quality control. The signals shorter than the 5-mers (designated as FITC) represent free FITC-12-ddUTP. See also Supplementary Fig. 4 for the procedure of enrichment of endo/lysosomal fraction. (Data are representative of two independent experiments). (**c**) Degraded dsDNA^FITC^ was detected in the cell lysates of Ramos Blue cells 24 h after cell treatment with dsDNA^FITC^. Input dsDNA^FITC^ and TCGTT^FITC^ alone were loaded for size and quality control. FITC signal was used to detect fragments of dsDNA^FITC^. The SYBR-Gold DNA staining was used for visualization of GeneRuler Ultra-Low-Range DNA ladder.

Because the effect of ODNs on TLR9 is augmented by sODNs, one would expect that ODN and sODN should colocalize in the endosomes. The cellular distribution of ODN and sODN was visualized in BM-DCs (Fig. 3a) and in HEK293 cells transfected with hTLR9 tethered to YFP (hTLR9^YFP^; Supplementary Fig. 3). Mouse-specific ODN1826 or human-specific ODN10104 and TCGTT, all labeled at the 3′-end, were used. The ODN1826 and sODN were colocalized in Rab5- and Rab7-positive endosomes 1 h post-addition (Fig. 3a). A similar distribution pattern of hODN10104 and sODN within the early and late endosomes was observed in HEK293 cells transfected with hTLR9 (Supplementary Fig. 3a). hODN10104 and sODN also colocalized with hTLR9^YFP^ (Supplementary Fig. 3b).

Furthermore, we enriched the endo/lysosomal fraction (Supplementary Fig. 4) and analyzed the presence of DNA content within these organelles (Fig. 3b). Magnetic separation was used for the enrichment of endo/lysosomes. HEK293 cells were fed with magnetic beads^21^,^22^ with gDNA. Endo/lysosomes that contained the magnetic beads were captured with a magnet and ruptured (Supplementary Fig. 4a), and their contents were analyzed for the presence of DNA fragments. The enriched endo/lysosomal fraction was positive for an early endosomal antigen, low for a succinate dehydrogenase complex, subunit A (SDHA) and actin (Supplementary Fig. 4b). Isolated short DNA was labeled with fluorescein isothiocyanate (FITC)-12-ddUTP using a recombinant shrimp alkaline phosphatase and a terminal deoxynucleotidyl transferase^23^ to facilitate detection. The enriched endo/lysosomes from cells fed with calf thymus gDNA contained mainly degraded gDNA, with the majority of fragments being shorter than 20 nt (Fig. 3b). Degraded DNA was also detected in the endo/lysosomal fraction of cells that were not fed DNA. These degraded DNA fragments likely originated from the degradation of autologous genomic and/or mitochondrial DNA^17^.

The FITC randomly labeled dsDNA (dsDNA^FITC^) was used to evaluate the degradation profile for dsDNA like bacterial DNA. In this instance the host DNA degradation products were not detected since only the degradation fragments of dsDNA^FITC^ were visible due to label. Similar to gDNA, the small fragments of degraded dsDNA^FITC^ were detected in cell lysates (Fig. 3c). Taken together, sODNs colocalize with ODNs in endo/lysosomes. We detected gDNA degradation products inside the enriched endo/lysosomes and degradation products of dsDNA within cell lysates, which may augment the activation of TLR9.

### sODN enhances the immune response to TLR9 agonists in mice

**Figure 4:**
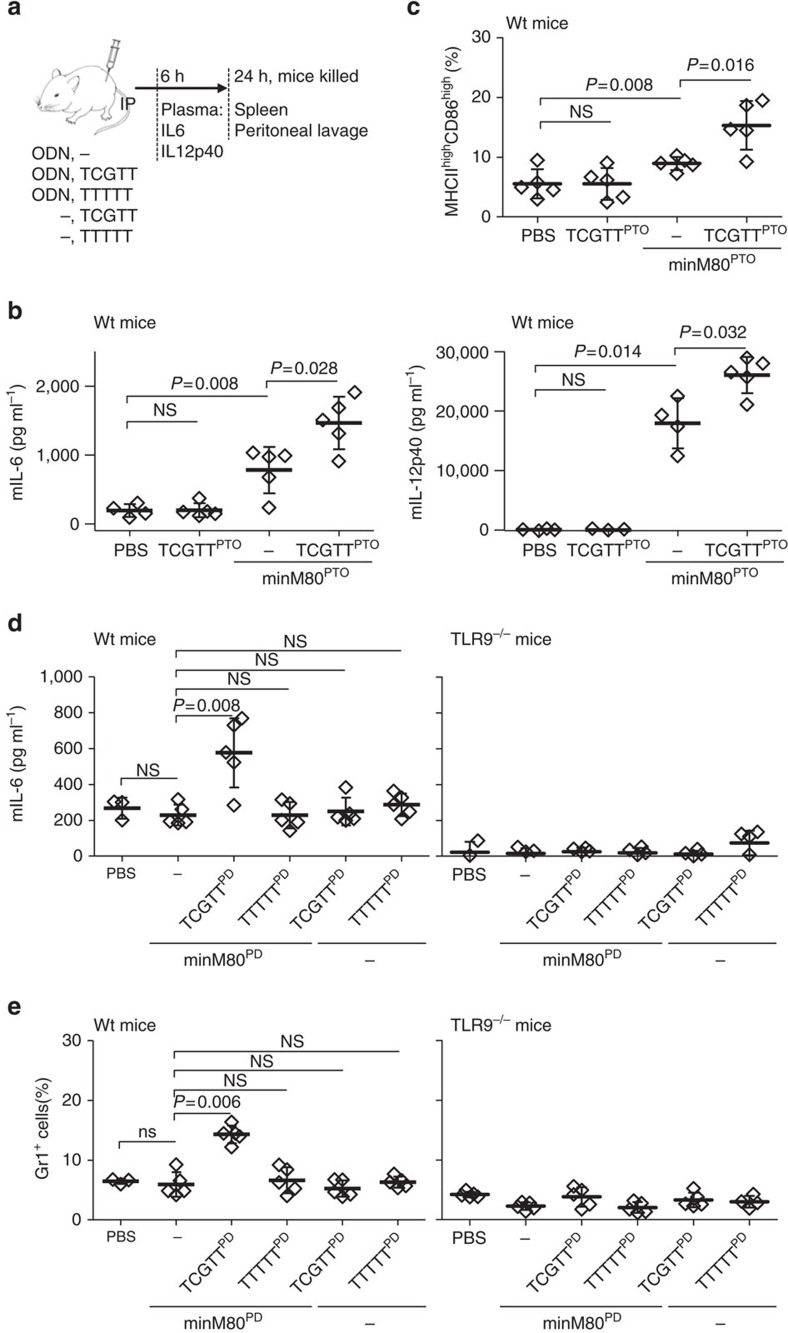
TCGTT augments the potency of ODN in the activation of TLR9-dependent innate immune response in mice. (**a**) A mice immunization protocol. (**b**,**c**) sODN^PTO^ with minM80^PTO^ promotes (**b**) the expression of mIL-6 and mIL-12p40 and (**c**) the migration of MHCII^high^ CD86^high^ cells into the peritoneal cavity. Mice were injected with PBS or minM80^PTO^ (40 nmol) with or without TCGTT^PTO^ (1 μmol). (**b**) Released mIL-6 and mIL-12p40 were measured 6 h after treatment. (**c**) Peritoneal lavage (at 24 h) was taken, and the cells were stained for cell surface markers MHCII and CD86 (Supplementary Fig. 6a presents the flow cytometer profiles). The percentages of MHCII^high^ and CD86^high^ cells for each mouse are given. (**d**,**e**) At suboptimal concentrations, ODN activates TLR9 response in mice only in the presence of TCGTT. Mice (wt and TLR9^−/−^) were treated with PBS or minM80^PD^ (120 nmol) with or without TCGTT^PD^ or TTTTT^PD^ (1.2 μmol) as a control. (**d**) Released mIL-6 was measured 6 h after treatment. (**e**) The infiltration of granulocytes (Gr-1-positive, CD20-negative and CD3ɛ-negative) into the spleens (at 24 h) of wt and TLR9^−/−^ mice was determined using flow cytometry (Supplementary Fig. 6b presents flow cytometer profiles). (Five mice per group were used, except for the PBS group, in which three (**d**) or five (**c**) mice were used. For each set, the mean and s.d. are indicated. A nonparametric Mann–Whitney test was used for the statistical comparison of the two samples; NS, *P*>0.05).

The physiological effect of CpG-containing sODNs on the modulation of TLR9 activation was further investigated in mice. The most pronounced effect on the part of TCGTT augmentation was observed when the basal degree of TLR9 activation with agonistic ODNs was weak (Supplementary Fig. 5a–e). Regardless of the ODN and sODN backbone, the potentiation of TLR9 activation with the combination of minH75 with sODN was significantly higher when low concentrations of ODN were used. This may be particularly important under subacute conditions, such as those caused by the release of endogenous DNA by injured cells, in which case the presence of sODNs can strongly promote TLR9 activation. Therefore, we designed two types of experiments activating inflammatory response in mice that differed in the initial activation of TLR9 with agonists: (i) one in which the activation of TLR9 was already achieved with immunostimulatory ODNs and (ii) another in which the amount of immunostimulatory ODNs was below the threshold level for the activation of TLR9. TCGTT^PTO^, minM80^PTO^, or a combination of TCGTT^PTO^ and minM80^PTO^ was injected intraperitoneally (IP) into mice (Fig. 4a). After 6 h, the mIL-6 and mIL-12p40 expression in the plasma was analyzed (Fig. 4b). We also identified the migration of MHCII^high^ CD86^high^ cells into the peritoneal cavity (Fig. 4c, Supplementary Fig. 6a,b). The injection of minM80 elevated the expression of cytokines and increased the percentage of infiltrating MHCII^high^ CD86^high^ cells. The addition of sODN to minM80^PTO^ significantly promoted cytokine expression (mIL-6, *P*=0.028; mIL-12p40, *P*=0.0014) and the number of MHCII^high^ CD86^high^ cells (*P*=0.016) in the peritoneal cavity. No changes were detected after treatment with sODN alone.

To determine whether sODN also boosts the activation of TLR9 at a subthreshold ODN concentration, mice were treated with minM80^PD^ at concentrations that did not trigger the detectable activation of TLR9 or with a mixture of minM80^PD^ and TCGTT^PD^ or TTTTT^PD^ to investigate the TCG motif requirement. In addition, TLR9^−/−^ mice were included to confirm that the observed effect depended on TLR9 (Fig. 4d,e and Supplementary Fig. 6c,d). Mice injected with TCGTT but not TTTTT triggered TLR9-dependent activation in the presence of minM80^PD^ (Fig. 4d). In addition, granulocyte infiltration into the spleen was triggered only by the mixture of minM80^PD^ with TCGTT^PD^, and not by the mixture of minM80^PD^ with TTTTT^PD^ or by minM80^PD^ or sODNs alone (Fig. 4e and Supplementary Fig. 6c,d). No granulocyte infiltration was detected in the TLR9^−/−^ mice. Taken together, the activation of the innate immune response with TLR9 agonists is potently augmented by sODNs in mice, which supports the role of sODNs as modulators of TLR9 activation.

### sODN induces the activation of hTLR9 by non-stimulating ODN

**Figure 5:**
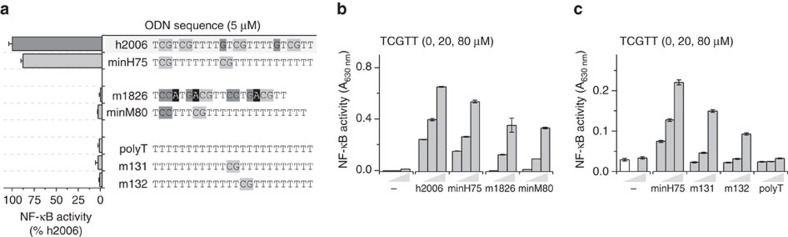
sODN promotes the activation of hTLR9 by ODNs containing single CpG motif. (**a**) ODNs with two CpG motifs activate hTLR9. Ramos Blue cells were stimulated with human-specific ODN2006^PD^ (h2006) and minH75^PD^, mouse-specific ODN1826^PD^ (m1826), minM80^PD^ and variants with PD backbones (all 5 μM). NF-κB/AP-1-dependent SEAP expression was analyzed 18 h after stimulation. Normalized SEAP expression was calculated relative to ODN2006 after the substraction of intrinsic activity. (**b**) sODN facilitates the activation of TLR9 with mouse-specific ODNs. (**c**) sODN also promotes the activation of hTLR9 for ODNs with one CpG motif, which were ineffective in activating hTLR9. Ramos Blue cells were stimulated with a mixture of ODNs^PD^ (2 μM) and sODNs^PD^. (**b**,**c**) NF-κB/AP-1-dependent SEAP expression was analyzed 18 h after stimulation. (Data are representative of three independent experiments. Bars represent the mean of five biological replicates ±s.d.).

sODNs with a TCG motif very efficiently augment the activation of TLR9 in combination with activating ODNs. Recently, we determined that two CpG motifs within the B-class ODN are required for the activation of hTLR9, while ODNs with a single CpG motif are able to activate mTLR9 but not hTLR9. We asked whether TCGTT would trigger the hTLR9 response in combination with ODN, which by itself does not activate TLR9. To test this hypothesis, we used ODN variants m131 and m132, which contain a single CpG motif, and mouse-specific ODNs that activate mTLR9 but not hTLR9 (refs 5, 6). As expected, m131, m132 and poly(T_20_) failed to activate hTLR9 (Fig. 5a). sODN TCGTT augmented the hTLR9 response in the presence of human-specific ODNs. Surprisingly, however, TCGTT triggered the activation of hTLR9 with mouse-specific ODNs, which on their own, were inactive (ODN1826 and minM80; Fig. 5b). Similarly, non-stimulating m131 and m132, in combination with TCGTT, activated hTLR9 (Fig. 5c). Poly(T_20_), an ODN lacking a single CpG motif, combined with TCGTT, had no impact on TLR9 activation. These data demonstrate that sODN promotes the activation of hTLR9 for ODNs with at least one CpG motif, which by themselves fail to activate TLR9. We conclude that TCGTT effectively substitutes for the requirement of an additional CpG motif in ODNs with only a single CpG motif in the activation of hTLR9.

### sODNs enhance response to mammalian gDNA and bacterial DNA

**Figure 6:**
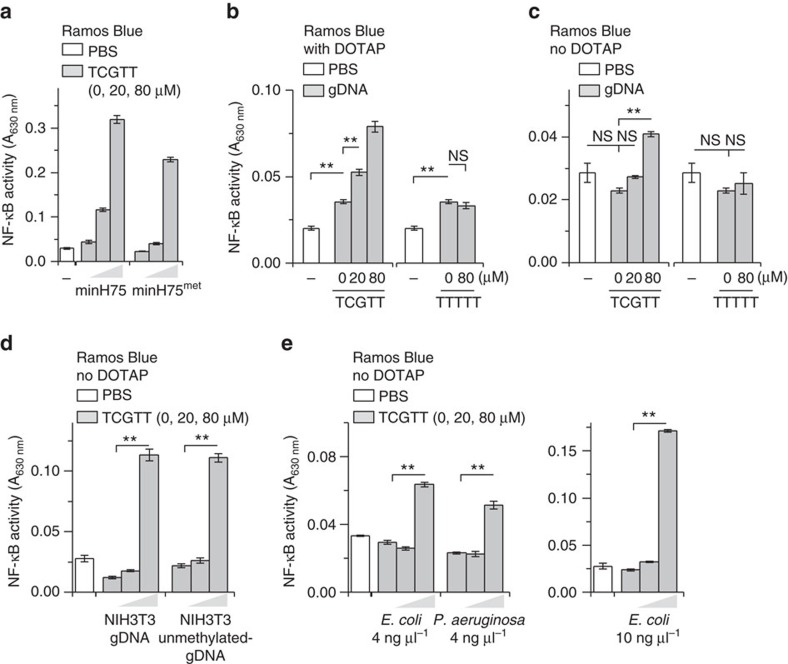
sODN enhances the activation of hTLR9 by eukaryotic and bacterial genomic DNA. (**a**) MinH75^met^ with methylated-CpG motifs activates hTLR9 in the presence of sODN. Ramos Blue cells were stimulated with a mixture of ODNs^PD^ (2 μM) and sODNs^PD^. (**b**–**d**) sODN TCGTT augments the activation of Ramos Blue cells stimulated with mammalian gDNA. Ramos Blue cells were stimulated with calf thymus gDNA (7.5 ng μl^−1^) with DOTAP (ratio DNA(μg):DOTAP(μl)=1:3) (**b**) or without DOTAP (**c**) in combination with TCGTT^PD^ or TTTTT^PD^. (**d**) Ramos Blue cells were stimulated with gDNA isolated from the mouse fibroblasts NIH3T3, or with PCR amplified gDNA from the NIH3T3 cells (unmethylated gDNA; 10 ng μl^−1^; without DOTAP). (**e**) The TLR9 activation with DNA isolated from bacteria was augmented by TCGTT. Ramos Blue cells were stimulated with DNA from bacteria *P. aeruginosa* (4 ng μl^−1^) and *E. coli* (4 and 10 ng μl^−1^) and TCGTT. (**a**–**e**) NF-κB/AP-1-dependent SEAP expression was analyzed 18 h later. (Data are representative of two independent experiments. Bars represent the means of five biological replicates ±s.d.; **P<0.05; NS, *P*>0.05, unpaired two-tailed Student's *t*-test).

TLR9 is activated by longer ODNs comprising a non-methylated CpG motif, while the methylation of a cytidine within a CpG motif blocks the activation of TLR9. However, sODN facilitates minH75^met^ ODN comprising methylated-CpG motifs in TLR9 activation (Fig. 6a), which may be important in the context of the recognition of mammalian gDNA with 80% methylated cytosines within the CG dinucleotides^24^,^25^,^26^.

Mammalian gDNA is a poor activator of hTLR9 due to the low incidence of the properly positioned double-CpG motifs and the high degree of cytosine methylation within the CG dinucleotides^27^. The unmethylated mammalian gDNA is better TLR9 agonist than mammalian gDNA^27^. We aimed to determine whether the activation of hTLR9 by mammalian gDNA may be enhanced in the presence of sODN (TCGTT). For comparison the unmethylated PCR amplified dsDNA, isolated from the mouse fibroblasts NIH3T3, was used. Two protocols were used to introduce mammalian gDNA to the cells (Fig. 6b–d; Supplementary Fig. 7a,b). Cells were stimulated with gDNA in the presence or absence of DOTAP, which facilitates the internalization of gDNA and protects gDNA from degradation with DNases^28^. Calf thymus gDNA and gDNA isolated from the mouse fibroblasts NIH3T3 was used. The weak activation of B-lymphocytes treated with gDNA in complex with DOTAP was observed (Fig. 6b). However, the addition of TCGTT to gDNA augmented TLR9 response in B-lymphocytes. TTTTT had no effect. Stimulation with gDNA in the absence of DOTAP had no effect on TLR9 activation. Similarly, the addition of TCGTT, but not TTTTT, induced hTLR9 activation by gDNA in the absence of DOTAP (Fig. 6c). Likewise, TCGTT potentiated the effect of gDNA on HEK293 cells transfected with hTLR9 or mTLR9 (Supplementary Fig. 7a,b). These data clearly show that sODN containing CG dinucleotide can potentially contribute to the activation of TLR9 by endogenous mammalian genomic DNA.

The CpG-containing ssDNA fragments originating from the degradation of bacterial and viral DNA are natural TLR9 ligands. Therefore, bacterial DNA isolated from *Pseudomonas aeruginosa* and *Escherichia coli* was tested for TLR9 activation. The TLR9 activation efficiency of *P. aeruginosa* and *E. coli* DNA correlates with CG content and number of CpG motifs^29^,^30^. The TCGTT augmented TLR9 activation with bacterial DNA regardless of DNA origin (Fig. 6e).

### Short ssDNA binds to TLR9

**Figure 7:**
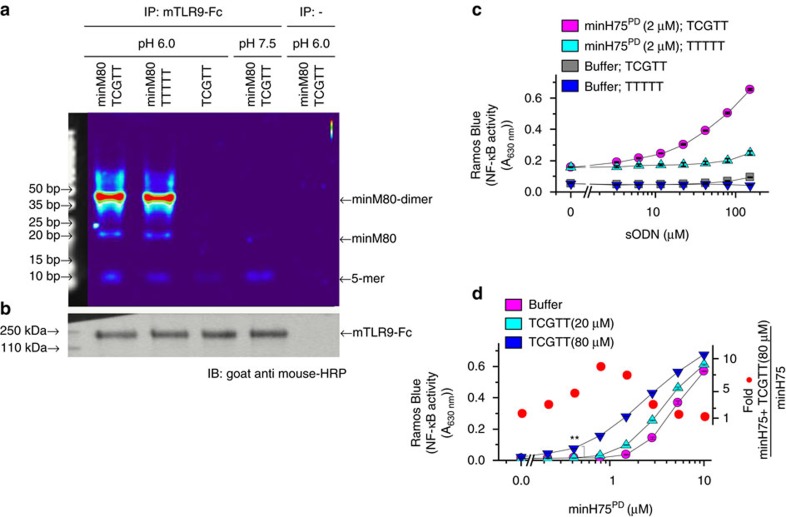
Binding of sODNs to TLR9 is independent of the CpG agonist binding site. (**a**) Immunoprecipitation was performed to assess the binding of sDNAs to mouse TLR9 at pH 6.0 and 7.5. ODN minM80^FITC^ (250 pmol) and/or sODNs (TCGTT^FITC^ or TTTTT^FITC^ (5 μmol)) were added to the protein-G-conjugated beads with bound mTLR9-Fc or control beads. The DNA fragments are visualized as an intensity heatmap. (**b**) mTLR9-Fc bound to Protein-G-beads was determined with western blot analysis using secondary goat anti-mouse-HRP antibody detection. See also Supplementary Fig. 8. (**c**,**d**) Dose response of hTLR9 activation for (**c**) sODNs (0-160 μM) at constant concentration of minH75^PD^ (2 μM) and (**d**) for minH75^PD^ (0-10 μM) at constant concentrations of TCGTT (20, 80 μM). Buffer or TTTTT were used instead of ODN and sODN as controls. Ramos Blue cells were stimulated with a mixture of ODNs^PD^ and sODNs^PD^. NF-κB/AP-1-dependent SEAP expression was analyzed 18 h later. Fold change in the TLR9 activity was calculated as ratio between SEAP expressions triggered by minH75 with TCGTT (80 μM) and minH75 alone. (Data are representative of two independent experiments. The means of three biological replicates ±s.d. are shown). See also Supplementary Fig. 9 for minM80^PD^ and ODNs with PTO backbone.

Based on the observed effect of sODNs on TLR9 activation, we surmised that it interacts with the ectodomain of TLR9. Indeed, immunoprecipitation experiments show the binding of TCGTT and minM80 to mTLR9-Fc at pH 6.0 (Fig. 7a,b, Supplementary Fig. 8a–d). TTTTT, in combination with minM80, and TCGTT alone were also able to bind to mTLR9-Fc. At pH 7.5, we detected some binding of TCGTT but no binding of minM80, even though the amounts of bound mTLR9-Fc to Protein-G-conjugated beads were comparable in all samples (Fig. 7b). The observed pH-dependent binding of ODN to mTLR9-Fc is in agreement with previous studies in which the *K*_d_ values for the binding of ODN1662_12mer to horse TLR9 were determined to be 500 times higher at pH 7.6 as compared to those at pH 6.0 (ref. 4). Based on results, we propose that sODN binds to a TLR9 dimer independent on binding of a stimulatory ODN.

Next we analyzed the dose response of hTLR9 activation for sODN at constant concentration of ODNs and for ODNs at constant concentration of sODN (Fig. 7c,d; Supplementary Fig. 9). The response of hTLR9 for the agonist minH75 and the minM80 was analyzed. The minM80 at tested concentrations failed to activate hTLR9. The augmented activation of hTLR9 correlated with increased concentration of TCGTT for both tested ODNs regardless the ODNs backbone. TTTTT expressed minor effect on augmentation of hTLR9 activity at higher concentrations of sODNs (Fig. 7c; Supplementary Fig. 9a). Those results suggest that the sODN binding site is probably not the primary CpG binding site of TLR9. If ODNs and sODNs were to compete for the same site, large concentrations of TCGTT would inhibit the TLR9 response. Furthermore, the activation efficacy of TLR9 was significantly enhanced with TCGTT at minH75^PD^ concentrations from 1 to 5 μM. At higher concentration of minH75^PD^, the effect of sODN was weaker suggesting that the sODNs stabilize the TLR9:ODN complex (Fig. 7d). Different response profile was observed for the minM80^PD^, which by itself is very poor activator of hTLR9, however, at high concentration of minM80 the significant enhancement of TLR9 activity was observed by the presence of a TCGTT (Supplementary Fig. 9b). A similar response was detected when PTO-based ODN was used instead of PD-based ODN (Supplementary Fig. 9c–f).

The internalization of ODNs to B-lymphocytes was not assisted or promoted by the sODNs (Supplementary Fig. 10a) as seen by chasing the internalization of FITC-labeled ODNs (ODN^FITC^) to Ramos Blue cells by flow cytometry. Furthermore, the cells were treated with the 5′- or 3′-FITC-labeled ODN^PD^ with or without TCGTT and 24 h later degradation of ODNs was analyzed within the cells (Supplementary Fig. 10b,c). The degradation of PD-based ODNs was not altered by sODNs.

Taken together, sODN binds to TLR9 independent of the presence of ODN and does not require acidic pH. The TCGTT had no effect on internalization of the agonist and its degradation. The TCGTT improves activation of TLR9 with ODNs, probably, by binding to TLR9 at the site different from the primary CpG binding site stabilizing active dimer.

## Discussion

Endosomal TLRs encounter a complex mixture of molecules that results from cellular sampling of the environment, endocytosis and the action of hydrolytic enzymes within those acidic compartments. The mixture of nucleic acid degradation products is particularly complex because they may be partially protected by nucleic acid binding proteins, which must be degraded by proteases. The degradation of microbial and autologous DNA generates a collection of ssDNA fragments^31^. Only a minority of ssDNA of suitable length also contains the minimal recognition motifs required to activate TLR9 (refs 5, 6). Surprisingly, we observed that TLR9 response is potentiated by short ssDNA fragments, sODNs, which by themselves, are unable to activate TLR9. We showed that even sODNs as small as 2 nt CG dideoxyribonucleotide augment the activation of TLR9; although the activation improvement was significantly lower as compared to the 3–5 nt sODNs comprising a 5′TCG motif. The TCG trinucleotide placed at the 5′-end of sODN exerted the most potent effect, regardless of the length of the sODN, similar to the impact of the TCG position within the minimal human-specific ODNs^5^. A bias of the CpG motif indicates that the binding of sODNs is sequence-dependent, much like TLR8, which binds the degradation products of RNA, UG diribonucleotide and uridine, all of which contribute to TLR8 activity^19^.

sODNs specifically augment TLR9 response with agonistic ODNs. Moreover, methylated-CpG-containing ODNs and CpG-containing ODNs, which by themselves fail to activate hTLR9 (refs 5, 32), did activate TLR9 in combination with sODNs.

Based on these results, we propose an extended model of TLR9 activation, which is particularly relevant for subtreshold amounts of immunostimulatory ODNs and activation by endogenous CG-methylated gDNA. Based on the crystal structure of the mTLR9-ODN complex, the 12 nt-long ODN binds to the mTLR9 ectodomain with a 2:2 stoichiometry^4^. The formation of active TLR9:ODN complex is most likely initiated by the binding of the ODN molecule to one of the ectodomains of the preformed inactive TLR9 dimer^33^, which should engage the binding sites of both ectodomains and reposition them into a favourable active conformation, which may, however, have a low stability. The binding affinity of sODNs for TLR9 must be weaker than that of the long. We propose that sODNs bind to a TLR9 at a site distinct from the CpG-immunostimulatory agonists binding site and strengthens the active dimer in the presence of the ODN ligand. The proposed extended model also provides an explanation for the activation of hTLR9 by ODNs comprising a single CpG motif (for example, mouse-specific ODNs), which due to their lack of the minimal motif requirement, fail to activate hTLR9. In recent studies, we determined that the minimal sequence requirements of the ODNs activating hTLR9 are more stringent in comparison to those activating mTLR9 (refs 5, 6). On the other hand, CpG-containing sODNs bypass the requirement of two CpG motifs in hTLR9. A similar effect on the part of sODN was also observed for ODNs with methylated cytosine within the CpG motifs. This effect may be particularly relevant to stimulation by mammalian gDNA. The frequency of the minimal hTLR9-stimulatory motifs is strongly underrepresented in the human genome, and ∼80% of the cytidine in the CG dinucleotides is methylated^24^,^25^,^26^. Our results, particularly the requirement of a CG dinucleotide within the sODN, and the binding study, support the notion that sODN directly interacts with the TLR9 ectodomain and augments its activation by ODN or non-stimulatory ODN. It is possible, however, that the interaction site is not the same as for stimulatory ODNs, much like the UG/U binding site of TLR8 (ref. 19) or the G binding site of TLR7 (ref. 20). We identified two regions important for TLR9 activation that overlap by location with the nucleoside binding site of TLR7 and TLR8. The Gln562, Gln346 and Arg348 contribute to the sequence-specificity recognition by hTLR9 in determining the bias for a pair of separated CpG motifs within the immunostimulatory ODNs^30^. This bias is lost in the presence of TCGTT.

The crystal structures of the activated forms of TLR7 or TLR8 complexed with natural agonists revealed that in addition to the ssRNA fragments both receptors also accommodate the nucleosides, UG/U for TLR8 and G for TLR7. The amino acid composition and location of the binding sites for the natural agonists are different between TLR7, TLR8 and TLR9 with a partial overlap between the position of ssRNA in the TLR7 dimer and the CpG-containing ODN in the TLR9 dimer. On the other hand, the amino acid residues forming nucleoside binding site are highly homologous between the TLR7 and TLR8. The nucleosides are embedded in the receptor dimerization interface bridging the two TLR ectodomains^19^,^20^.

Similar to sODNs (TCGTT) and TLR9, guanosine or uridine alone cannot effectively activate TLR7 or TLR8, respectively, in the cellular assay^34^. Only in combination with the natural ligand, nucleosides (at 1 mM) can potentiate the activation of TLR7/TLR8. That is probably due to the improved binding of nucleosides which is stronger in the presence of the polyU for TLR7 (*K*_d_ approx. 1.5 μM) and the ORN06 for TLR8 (*K*_d_ approx. 1 μM)^19^,^20^. Whether the nucleosides redefine a ‘loose' RNA sequence specificity for the TLR7 and TLR8 as the TCGTT for hTLR9 has not been analyzed.

Several TLRs comprise multiple binding sites for the agonist. Two pseudosymmetric binding sites on the TLR3 ECD recognize the two sites of the dsRNA duplex^35^,^36^,^37^,^38^. TLR8 binds the uridine and UG dinucleotide of the ssRNA^19^, TLR5 binds flagellin across its entire D1 domain^39^ and two sites of TLR4 bind the complex composed of the coreceptor MD-2 and LPS with a 2:2:2 stoichiometry^40^.

It is likely that the endosomes contain a substantial number of sODNs. However, the strongly augmented response due to the addition of sODN indicates that they may represent the limiting amount for signalling. On the other hand, the presence of cfDNA due to tissue injury or microbial replication may represent a mechanism for controlling TLR9 activation. In healthy individuals, the level of autologous cfDNA in the plasma is ∼0.1 μg ml^−1^. An elevated level of plasma cfDNA is a biomarker for pathological conditions^41^,^42^,^43^, being significantly increased in cancer^44^, trauma^45^, diabetes^46^, autoimmune diseases^47^ and infection^48^. In cell cultures, a dramatic increase in the concentration of cfDNA was determined after the induction of apoptosis and necrosis^49^. It is difficult to determine the amount of endosomal sODNs after the physiological endocytosis of microbes or immune complexes, but it is clear that the molecular species of DNA degradation products within endosomes are diverse. DNA in microbes is protected from DNases and is released only after microbial lysis^9^,^50^, which takes place in the endosomes. Similarly, DNA from apoptotic cells is protected by histones^51^ and is mainly degraded on endocytosis^15^. In necrotic cells, on the other hand, DNase I, in concert with proteases, removes nucleosomes, resulting in DNA fragmentation^52^. A discrete 10-nt DNA fragment profile is characteristic of DNase I-processed nucleosomes^53^, and fragments longer than 3 nt are generated from unprotected DNA by DNase I (ref. 54). The four terminal 3′ nucleotides of paired oligonucleotides are completely resistant to cleavage by DNase II, generating fragments longer than 4 nt (ref. 54). Therefore, the presence of short DNA fragments is expected within endosomes.

In addition, sODNs significantly enhanced TLR9-dependent inflammatory response in mice, which can be a driver or initiator of diseases when coupled with inflammation. Excessive and persistent NETs (combining microbial and autologous DNA degradation products) have been shown to be involved in the pathogenesis of diverse disorders, including autoimmune diseases and impaired wound healing^42^,^55^. In liver injuries, hepatocytes can release dsDNA, and TLR9 sustains liver diseases due to the recognition of extracellular DNA after necrotic cell death^56^. Autologous and microbial DNA degradation products are involved in continuous immune-cell stimulation, assisting autoimmune diseases^57^ and diabetes^58^.

While it remains to be determined how sODNs participate in the formation of active TLR9, our study provides new insights regarding the activation mechanisms of TLR9 and emphasizes the importance of cfDNA and its short degradation products as players in activating TLR9-dependent inflammatory response. Finally, the decrease of the threshold for TLR9 activation may be relevant to therapeutic applications in that the targeted delivery of immunostimulatory ODNs may have more potent effect, while it would not activate other cells.

## Methods

### Cell lines and mice

Human embryonic kidney cell line HEK293 (ATCC CRL-1573) was cultured in complete media (Dulbecco's Modified Eagle Medium (DMEM); 1 g l^−1^ glucose, 10% heat-inactivated fetal bovine serum (FBS; Gibco, Invitrogen)) in 5% CO_2_ at 37 °C. B-lymphocytes (Ramos Blue cells (InvivoGen)), which stably express an NF-κB/AP-1-inducible SEAP reporter gene, were cultured in Iscove's Modified Dulbecco's Medium (IMDM; Gibco, Invitrogen) supplemented with 10% (v/v) heat-inactivated FBS and zeocin (100 μg ml^−1^) at 37 °C in 5% CO_2_. HEK-Blue hTLR9, HEK-Blue mTLR9 or HEK-Blue Null 1 (all InvivoGen) cells were cultured in complete media (DMEM; 1 g l^−1^ glucose, 10% heat-inactivated FBS) supplemented with zeocin (100 μg ml^−1^ all HEK-Blue cells) and blasticidin (10 μg ml^−1^ for HEK-Blue hTLR9 and HEK-Blue mTLR9).

Wt C57BL/6 OlaHsd mice and B6.129-Tlr9^tm1Aki^ mice were purchased from Harlan (Italy) and Ifrec (Japan), respectively. Eight- to 13-week-old male and female mice were used for the experiments. All of the procedures involving animals were performed according to the directives of the EU 2010/63 and were approved by the Administration of the Republic of Slovenia for Food Safety, Veterinary, and Plant Protection of the Ministry of Agriculture, Forestry, and Foods, Republic of Slovenia (Permit no. U34401-37/2015/5).

### Plasmids and reagents

Expression plasmids containing human TLR9 gene (pUNO-hTLR9^HA^) and mouse TLR9 (pUNO-mTLR9^HA^) were obtained from InvivoGen. Plasmid phRL-TK constitutively expressing *Renilla* luciferase for the normalization of transfection efficiency was obtained from Promega. The plasmid coding for firefly luciferase under NF-κB promoter (pELAM-1-luciferase) was a gift from C. Kirschning (Institute for Medical Microbiology, University of Duisburg-Essen, Essen, Germany).

The cells were treated with various TLR9 agonists: human-specific ODN2006 (hODN2006) and minH75 (minH)^5^, mouse-specific ODN1826 (mODN1826) and minM80 (minM)^6^ (B-class) with a phosphorotioate (PTO) or PD backbone. The other minimal ODNs had PD backbones (all oligos from Integrated DNA Technologies). C-class ODN2395 and A-class human-specific ODN2216 and mouse-specific ODN1585 with a PD backbone were used. The PTO- or PD-based sODNs of 5 nt and longer were obtained from Integrated DNA Technologies, while the shorter sODNs (2-4 nt) were obtained from Eurogentec. Genomic calf thymus DNA (gDNA) was obtained from Sigma. The genomic DNA was mixed with DOTAP (Roche; cat. no. 11202375001) at a 1:3 ratio (1 μg DNA, 3 μl DOTAP).

gDNA from the mouse fibroblasts NIH3T3 (ATCC CRL-1658) was isolated using DNeasy Blood and Tissue Kit (QIAGEN; cat. no. 69506) according to manufacturer's instructions. The unmethylated DNA was obtained by two-step PCR amplification of NIH3T3 gDNA using random hexameter primers (IDT, ReadyMade Randomers; cat. no. 51-01-18-26). For one reaction (25 μl), we used 120 ng of gDNA, 20 pmol μl^−1^ primers, 12.5 μl of 2 × KAPA HiFi HotStart ReadyMix (Kapa Biosystems) and 1 μl of DMSO. The PCR regimen was: 95 °C for 3 min; 98 °C for 1 min, 11 °C for 1 min and 72 °C for 90 s (35 times); and 72 °C for 5 min. After 35 cycles, 25 μl of fresh reaction mix without DNA was added (resulting in 50 μl final volume), and 45 additional cycles were run. The PCR regimen was 95 °C for 2 min; 98 °C for 30 s, 11 °C for 30 s and 72 °C for 90 s (45 times); and 72 °C for 5 min (ref. 30). DNA concentration was determined with Quant-it dsDNA Assay Kit (high sensitivity, Invitrogen; cat. no. Q33120) according to manufacturer's instructions.

FITC-labeled dsDNA (dsDNA^FITC^) was obtained by PCR amplification of DNA fragment from mTLR9 (template pUNO-mTLR9^HA^; primers: 5′-GCCGTTACAGATCCAAGCTG-3′; 5′ CTGCATTCTAGTTGTGGTTTG-3′) with FITC-12-dUTP nucleotides (PerkinElmer) resulting in 3244, bp long fragment. Fluorescently labeled DNA was produced by two-step PCR using specific primers and DreamTaq PCR Master Mix (Thermo Scientific). For one reaction (25 μl) we used 20 ng of plasmid DNA, 20 pmol μl^−1^ of each primer, 12.5 μl of 2 × DreamTaq PCR Master Mix and 50 μM FITC-12-dUTP (ratio 1:4 of FITC-dUTP:dTTP). The PCR regimen was: 95 °C for 3 min; 95 °C for 30 s, 53 °C for 30 s, and 72 °C for 190 s (40 times); and 72 °C for 5 min. After 40 cycles, the fresh reaction mix (without DNA template) was added (resulting in 50 μl of final volume) and another 45 cycles were run. PCR fragments were cleaned using GeneJET PCR Purification Kit (Thermo Scientific; cat. no. K0701).

### Luciferase reporter assay

HEK293 cells were harvested from an actively growing culture and plated onto CoStar White 96-well plates (Corning) at 2.2 × 10^4^ cells per well (0.1 ml). After 24 h at 50% confluence, the cells were transiently transfected with plasmids expressing wt TLR9 (20 ng DNA per well) or pcDNA3 (Invitrogen), UNC93B1 (5 ng DNA per well), ELAM1-luciferase reporter plasmid (50 ng DNA per well), and phRL-TK (5 ng DNA per well, unless stated otherwise) using Lipofectamine 2000 according to the manufacturer's instructions (Invitrogen). After 24 h, the culture medium was replaced with fresh medium, and the cells were stimulated with TLR9 agonists for 18 h. The cells were lysed in passive lysis buffer (Promega) and analyzed for reporter gene activities using a dual-luciferase reporter assay. Error bars represent the s.d. obtained from at least three biological replicates.

### SEAP reporter assay

Ramos Blue cells, HEK-Blue hTLR9, HEK-Blue mTLR9 or HEK-Blue Null 1 cells were seeded at a density of 2 × 10^5^ cells per well, 1 × 10^5^ cells per well, 0.5 × 10^5^ cells per well or 0.7 × 10^5^ cells per well, respectively, onto the 96-well plates (TPP). The cells were immediately stimulated with ODNs (final concentrations included in graphs). After 18 h, the supernatants were collected, and NF-κB/AP-1 activation linked to SEAP was determined using a Quanti Blue reagent according to the manufacturer's instructions (InvivoGen; cat. no. rep-qb2). Error bars represent the s.d. obtained from at least three biological replicates.

### Detection of DNA degradation in endosomes

The protocol for the isolation of endosomes was adapted from Scheffer *et al*.^59^, Wittrup *et al*.^60^, Chen *et al*.^61^, Li *et al*.^62^ and Schröter *et al*.^63^. HEK293 cells were seeded on a 6-cm plate (TPP) at a density of 1.0 × 10^6^ cells per plate and cultured in DMEM medium with 10% FBS depleted of exosomes. The next day, the medium was replaced with 4 ml of fresh DMEM supplemented with Pen/Strep (1:100) and 0.5 mg ml^−1^ BSA. The mixture of latex magnetic beads (7.4 mg ml^−1^)^21^,^22^ and DNA (800 μg of calf thymus DNA) or magnetic beads alone were added. After 16 h, organelles with internalized magnetic beads were isolated.

The cells were washed five times with ice-cold PBS (4 ml) and detached with a cell scraper in 4 ml of PBS. The cells were collected via centrifugation at 1,200 r.p.m. and 4 °C 5 min and resuspended in 1 ml of homogenization buffer (10 mM Hepes, pH 7.2, 100 mM KCl, 1 mM EDTA, 250 mM sucrose, 0.1 mg ml^−1^ RNaseA (Fermentas) and protease inhibitors (Roche Complete mini)). The cells were mechanically disrupted via passage through a 27G needle 20 times. Organelles containing magnetic beads were captured using a magnetic stand (Millipore) and washed four times with homogenization buffer to remove the remaining intact cells, cell debris and nonmagnetic organelle fraction. The magnetic organelles were resuspended in 50 μl of hypotonic lysis buffer (10 mM Tris-HCl, pH 7.5, 1% Triton-X100), incubated on ice for 20 min, and then frozen and thawed four times in liquid nitrogen. Finally, the supernatant, with endosomal content, was collected with centrifugation for 10 min at 30,000 RCF (4 °C) and stored at −80 °C.

For the visualization of the short DNA fragments, the DNA from the enriched endo/lysosomes was labeled with FITC-12-ddUTP (PerkinElmer). The total protein and DNA concentrations in these supernatants were measured. Fractions containing 3 μg of total proteins were used in experiment. The amount of DNA in these fractions was ∼5 pmol for the sample with the added calf thymus DNA and ∼1.6 pmol for the sample without the added DNA. Before labeling with FITC-12-ddUTP, the 3′-ends of the DNA were dephosphorylated with recombinant shrimp alkaline phosphatase (rSAP; NEB; cat. no. M0371S) according to the manufacturer's instructions. Briefly, 5 pmol of DNA was treated with rSAP in CutSmart Buffer for 30 min at 37 °C. The reaction was stopped via incubation at 65 °C for 10 min. Subsequently, dephosphorylated DNA or ODNs (minM80^PD^ and TCGTT^PD^) were incubated with 8U of terminal deoxynucleotidyl transferase (TdT, NEB; cat. no. M0315S) and 0.05 mM FITC-12-ddUTP at 37 °C for 2 h and subsequently mixed with formamide loading buffer (22.6 M formamide, 11.1 μM EDTA pH 8) and heated to 95 °C for 5 min. Labeled nucleic acids and standard (GeneRuler Ultra-Low-Range DNA Ladder, Thermo Scientific) were separated using 20% TBE-urea polyacrylamide gel in TBE buffer at 200 V for 20 min. The FITC-labeled DNA was imaged using IVIS Lumina (PerkinElmer). Subsequently, the gels were stained for 20 min with SYBR Gold Nucleic Acid Gel Stain (Invitrogen: cat. no. S11494), diluted in TBE buffer (1:5,000), and imaged using IVIS Lumina (to detect the standard).

Proteins from the isolated organelles were separated via SDS–polyacrylamide gel electrophoresis (SDS–PAGE), transferred to Hybond ECL nitrocellulose membrane (GE Healthcare), and incubated with primary antibodies: mouse monoclonal anti-β-actin (1:2,000; 3700, Cell Signaling), rabbit polyclonal anti-endosomal early antigen (1:1,000, ab2900, Abcam), rabbit polyclonal anti-succinate dehydrogenase, protein A (SDHA, 1:1,000, 11998P, Cell Signaling), followed by incubation with secondary goat anti-mouse IgG-HRP (sc-2005, Santa Cruz) and goat anti-rabbit IgG-HRP (ab6721, Abcam) antibodies (both diluted 1:4,000). Secondary antibodies were detected with the ECL Western blotting detection reagent (GE Healthcare), according to the manufacturer's protocol.

### Detection of DNA degradation in whole-cell lysates

The Ramos Blue cells were seeded at a density of 1 × 10^6^ cells per well (500 μl) onto the 24-well plates (TPP). The cells were treated with minM80^PD-5′FITC^ (5 μM) or dsDNA^FITC^ (16.8 ng μl^−1^) with or without TCGTT^PD^ or TTTTT^PD^ (80 μM). Eleven or 24 h later cells were collected, washed 4 times with PBS and resuspended in formamide loading buffer and incubated at 95 °C for 10 min. Lysates were frozen (−80 °C) and again incubated at 95 °C for 10 min. The protein content was measured with BCA test (Sigma). Lysates were separated using 20% TBE-urea polyacrylamide gel in TBE buffer at 200 V for 30 min. To ensure equal loading the amount of each lysate was normalized to equal protein content. The labeled DNA was imaged using IVIS Lumina. Subsequently, the gels were stained for 20 min with SYBR Gold Nucleic Acid Gel Stain, diluted in TBE buffer (1:5,000), and imaged using IVIS Lumina (to detect the standard). Each experiment was performed in three biological replicates.

### Cytokine detection from mouse BM-DCs

Mouse bone marrow (BM) was obtained by flushing the femurs and tibiae taken from 8 to 10-week-old wt C57BL/6 OlaHsd and B6.129-Tlr9^tm1Aki^ (TLR9^−/−^) mice with RPMI-1640 and 2% FBS. After the lysis of the red blood cells with RBC buffer (red blood cell lysis buffer, Biolegend) for 5 min on ice, the cells (2 × 10^6^ cells ml^−1^) were cultured at 37 °C for a week in media containing RPMI-1640, 10% FBS, 2 mM L-glutamine, penicillin (100 U ml^−1^), streptomycin (0.1 mg ml^−1^), IL4 (5ng ml^−1^) and mGMC-SF (10 ng ml^−1^) (for BM-DC^GMC-SF,IL4^). At day 3, an equal volume of fresh media was added to the cells. At day 6, immature BM-DC were harvested. The cells were seeded onto 96-well round bottom plates- (Corning; 1 × 10^5^ cells per well in 100 μl). The cells were stimulated with ODNs (concentrations indicated on graphs). Mouse (m)IL-6 secreted in the media was determined with ELISA (mIL-6 Elisa Ready-Set-Go, eBioscience). Error bars represent the s.d. values obtained from at least three biological replicates.

### Confocal imaging analysis

HEK293T and BM-DC^GMC-SF, IL-4^ (BM-DC) cells were seeded onto eight-well tissue culture chambers (Ibidi) at 2 × 10^5^ cells per well or 6 × 10^5^ cells per well, respectively. After 24 h, the HEK293T cells were transfected with the plasmids encoding TLR9^YFP^ or TLR9 (140 ng DNA per well). The 24 h post transfection, the cells were treated with 5 μM TCGTT^PD-3′Cy3^ and 3 μM human-specific ODN10104^PD-3′Cy5^. After 18 h, the cells were fixed (Histofix, Roth; 4% formaldehyde) and permeabilized (0.1% Triton-x100, Sigma). The cells were stained for Rab5 and Rab7 proteins with anti-Rab5^ATTO390^ and anti-Rab7^AlexaFluor488^ antibodies (1:100, ABIN2486165 and ABIN912733, respectively, Antibodies Online).

Twenty-four hours post-seeding, the BM-DC^GMC-SF, IL-4^ (BM-DC) cells were treated with 5 μM TCGTT^PD-3′Cy3^ and 3 μM mouse-specific ODN1826^PD-3′Cy5^. After 1 h, the cells were fixed and permeabilized. The cells were again stained for Rab5 and Rab7 proteins with anti-Rab5^ATTO390^ and anti-Rab7^AlexaFluor488^ antibodies.

Images were acquired using the Leica TCS SP5 inverted laser-scanning microscope on a Leica DMI 6000 CS module equipped with a HCX Plane-Apochromat lambda blue × 63 oil-immersion objective with NA 1.4 (Leica Microsystems). The images were processed with LAS AF software (Leica Microsystems) and ImageJ software (National Institute of Mental Health, Bethesda, MD, USA).

### Animal experiments

Eight to 13-week-old wt C57BL/6 OlaHsd and B6.129-Tlr9^tm1Aki^ (TLR9^−/−^) mice were injected IP with PBS and minM80^PD^ (120 nmol per mouse) alone or combined with TCGTT^PD^ or TTTTT^PD^ (both 1,200 nmol per mouse) and with TCGTT^PD^ or TTTTT^PD^ alone. In another test, 8–13-week-old wt C57BL/6 J (TLR5^−/−^) mice were injected IP with PBS and m80^PTO^ (40 nmol per mouse) alone or combined with TCGTT^PTO^ (1,000 nmol per mouse) and with TCGTT^PTO^ alone. In both experiments, blood was first taken after 6 h. After 24 h, blood was collected and afterwards the mice were humanely sacrificed with cervical dislocation. Peritoneal lavages were prepared via the injection of 6 ml of ice-cold PBS into the peritoneal cavity, resulting in 4–6 ml of lavages, and the spleens were removed. A nonparametric Mann–Whitney test was used for the statistical comparison of the two experimental conditions.

### Cytokine detection from mouse blood

Blood samples were collected in heparinized tubes (Sarsted). Aliquots of plasma were prepared by centrifugation at 3,000 r.p.m. at 4 °C for 30 min and kept at −80 °C until assayed for mIL-6 and mIL-12p40 using ELISA kits (mIL-6, mIL-12p40 ELISA Ready-Set-Go, eBioscience).

### Flow cytometry

Single-cell suspensions were prepared from spleens and subsequently treated with RBC buffer (red blood cell lysis buffer, Biolegend). The cells were stained with antibodies against GR-1, CD20 and CD3ɛ. The cells from the peritoneal lavages were stained for CD11b, MHCII and CD86. Antibodies for anti-mouse Ly-6G (Gr-1) FITC (clone 1A8-Ly6g), anti-mouse CD20-PE (clone REA294), anti-mouse CD3ɛ-PE-Vio770 (clone 17A2), anti-human and -mouse CD11b-APC (clone M1/70.15.11.5) and anti-mouse MHCII-VioBlue (clone M5/114.15.2) were obtained from Miltenyi Biotec and were diluted 1:100. Anti-mouse CD86 (B7-2) PE (clone PO3.3) was from eBioscience. 1:320 dilution was used. Together with antibodies, FcR blocking reagent (FcR Blocking Reagent mouse, Miltenyi Biotec) was added (1:100 dilution). Cells were incubated for 30 min at 4 °C. Subsequently, all cells were fixed with Histofix (Roth) reagent. The cells were analyzed on flow cytometer (CyFlow space, Partec). Data were analyzed with FlowJo software (Tree Star).

### Immunoprecipitation binding assay

For the binding studies, a soluble mTLR9 ectodomain fused to the Fc fragment of mouse IgG (mTLR9-Fc, R&D Systems) was bound to protein-G-coupled Dynabeads (Dynabeads Protein G for Immunoprecipitation, Novex; cat. no. 10003D). Briefly, 7.5 μg of mTLR9-Fc (or PBS alone—negative control) was incubated with 70 μl of beads for 3 h at room temperature and washed three times with wash buffer (1 × PBS, 0.02% Tween-20, pH 7.5 or pH 6.0). Then, minM80^PD-3′FITC^ (250 pmol) and/or TCGTT^PD-3′FITC^ or TTTTT^PD-3′FITC^ (5 μmol) in phosphate buffer (pH 6 or 7.5) were added per sample, incubated for 3 h at room temperature, and washed three times with wash buffer of an appropriate pH. Bound DNA and mTLR9-Fc were eluted via incubation at 95 °C for 10 min in 42 μl formamide loading buffer. The ODNs and standard were separated using 20% TBE-urea polyacrylamide gel in TBE buffer and imaged using an IVIS Lumina (PerkinElmer). Then, mTLR9-Fc was detected with western blot analysis using goat anti-mouse-HRP antibodies (1:4,000, Santa Cruz Biotechnologies, sc-2005).

### Internalization of ODN

The Ramos Blue cells were seeded at a density of 1 × 10^6^ cells per well (500 μl) onto the 24-well plates (TPP). The cells were treated with minM80^PD-5′FITC^ (2 μM) without or with TCGTT^PD^ or TTTTT^PD^ (80 uM). Immediately after addition (0 h) and 2 h, and 4 h later, samples (50 μl) were taken; the cells were collected, washed and resuspended in FACS buffer (PBS, 0.5% BSA). Internalization of FITC-labeled ODN was analyzed by flow cytometry. Each experiment was performed in three biological replicates.

### Software and statistics

Graphs were prepared with Origin 8.1 (http://www.originlab.com/), and GraphPad Prism 5 (http://www.graphpad.com/) was used for statistical purposes. An unpaired two-tailed *t*-test (equal variance was assessed with the F-test assuming normal data distribution) or nonparametric Mann–Whitney test (animal experiments) was used for the statistical comparison of the data. The Mead's resource equation was used to estimate the sample size of laboratory animals. A calculated degree of freedom (*E*) was 15 and 20 (no. of animals 20 and 25 and 5 animals per group).

### Data availability

The authors declare that the data supporting the findings of this study are available within the paper and its Supplementary Information files or are available from the corresponding author upon reasonable request.

## Additional information

**How to cite this article:** Pohar, J. *et al*. Short single-stranded DNA degradation products augment the activation of Toll-like receptor 9. *Nat. Commun.* **8,** 15363 doi: 10.1038/ncomms15363 (2017).

**Publisher's note:** Springer Nature remains neutral with regard to jurisdictional claims in published maps and institutional affiliations.

## Supplementary Material

Supplementary Information Supplementary Figures

Peer Review File
