# Supplementary material for: Short single-stranded DNA degradation products augment the activation of Toll-like receptor 9
Source: Nat Commun. 2017 May 22;8:15363. doi: 10.1038/ncomms15363 (PMC5458134; doi:10.1038/ncomms15363)
Supplement: Supplementary Information — Supplementary Figures [file ncomms15363-s1.pdf]

Reviewers' comments:

Reviewer #1 (Remarks to the Author):

The manuscript by Pohar et al. report that 5base short single-stranded DNA deoxyoligonucleotides with and a TCGTT motif augment toll-like receptor 9 activation.
The study is of interest since such short DNA degradation products generated in endosomes from genomic DNA augment TLR9 activation and may impact on immune responses to infection or autoimmune disease.
1) The experiments describing the TCG-dependency on augmentation on TLR9 activation in vitro and in vivo are very convincing (Fig. 1, 2 and 4). However , the effect is only seen at quite high concentrations such as 80µM in vitro or 2mg per mouse in vivo. The authors do not really discuss why that is the case? My first thought was that the added short ODN protects the stimulatory ODN from DNase degradation and inactivation. In this scenario, the corresponding DNase may have some specificity for the TCG motif. The authors should elaborate on this issue.
2) Fig. 3 B is not convincing or misunderstood by the reviewer. The authors claim that DNase digestion products are enriched in the endosome. However, the gDNA input control that was loaded on the gel as control, already contains similar small fragments as the DNA isolated from endosomes. What is the proof for fragment generation in the endosome.
3) The binding studies of short ODNs with mouse TLR9-Fc does not show any binding specificity for the short ODN with the CG motif (Fig. 7A), sce it also binds TTTTT ODNs. Shouldn't according to the authors view, the TCG bind better (or exclusively)? The quality of the blot in Fig 7B is of poor quality and I was unable to judge it.
4) In the discussion the authors could elaborate more on the similarities (and differences) on TLR8 and TLR9 structure in regard to binding pockets for small CG containing fragments.

Overall, the manuscript is interesting, but falls short on some issues (see above). Therefore I would recommend "Major Revision" of the manuscript.

Reviewer #2 (Remarks to the Author):

In the manuscript entitled 'Short single-stranded DNA degradation products augment the activation of Toll-like receptor 9' Pohar and colleagues report the observation, that short CG containing DNA can increase the response of TLR9 towards longer oligonucleotides.
This observation is novel and worth reporting and overall topic is of interest to the readership of Nature Communications. Overall I am supportive of publishing this article.
I have a conceptual issue that needs to be addressed. The TLR9/ODN co-crystal structure with immune stimulatory ODNs shows a 12 nucleotide ODN binding to TLR9 in a 2:2 stoichiometry. Importantly, the structure demonstrated that the integrity of the ODN is important since one binding side on one TLR9 recognizes the CpG motif, while another binding site on the dimerization partner recognizes the ODN backbone. I think what the authors wish to claim regarding the putative mechanism how the short ODNs function is misleading. They speculate that short ODNs bind to a TLR9 dimer that is already crosslinked by a single stimulatory ODN and that thereby the short ODNs would enhance TLR9 activation only if sub-optimal ligand concentration of the longer ODN is present. If such a model is correct, then larger amounts of the sODNs should even inhibit the responses to long ODN, because the ODNs compete for the same binding site. This mode would be different from the cooperative activation mode of TLR8 where two binding sites are postulated, one of which acts

allosterically.
The authors should address this point experimentally to work out the mechanisms in more detail. Otherwise the paper stays very descriptive.
Another suggestion to make the observation reported in this paper more relevant is to study the ODN sizes appearing upon viral infection and/or to study whether sODNs would augment the responses towards natural TLR9 stimuli.

REVIEWERS' COMMENTS:

Reviewer #1 (Remarks to the Author):

The authors convincingly addressed my concerns. The added experiments strongly supoort the authors claims.
Therefore I would recommend publishing of the manuscript.

Reviewer #2 (Remarks to the Author):

The authors have responded to the reviewer's critique appropriately and have provided new experimental data for most points raised. My questions I had were addressed satisfactorily.
I am in favor of publishing the paper in its current version.

Reviewers' comments:

Reviewer #1 (Remarks to the Author):

The manuscript by Pohar et al. report that 5 base short single-stranded DNA deoxyoligonucleotides with and a TCGTT motif augment toll-like receptor 9 activation.

The study is of interest since such short DNA degradation products generated in endosomes from genomic DNA augment TLR9 activation and may impact on immune responses to infection or autoimmune disease.

1.1) The experiments describing the TCG-dependency on augmentation on TLR9 activation *in vitro* and *in vivo* are very convincing (Fig. 1, 2 and 4). **However, the effect is only seen at quite high concentrations such as 80 µM in vitro or 2 mg per mouse in vivo. The authors do not really discuss why that is the case?** My first thought was that the added short ODN protects the stimulatory ODN from DNase degradation and inactivation. In this scenario, the corresponding DNase may have some specificity for the TCG motif. **The authors should elaborate on this issue**.

In order to respond to the issue raised by the reviewer we focused on two points to clarify the possible effect of TCGTT in degradation of TLR9 agonists. First, the role of TCGTT as a protective agent against degradation of the agonistic ODN was analyzed. We treated cells with the minM80 labeled with FITC (minM80$^{PD_5'FITC}$) without and with sODNs (**Fig 1.1A**).No difference in the size of the minM80 $^{PD\_5'FITC}$ was observed without or with sODNs.

To exclude the effect of TCGTT on the internalization, cells were treated with the FITC-labeled agonist without and with TCGTT and internalization of the agonist was followed by the flow cytometry (**Fig 1.1B**). This demonstrated that sODN have no impact on the internalization of ODN.

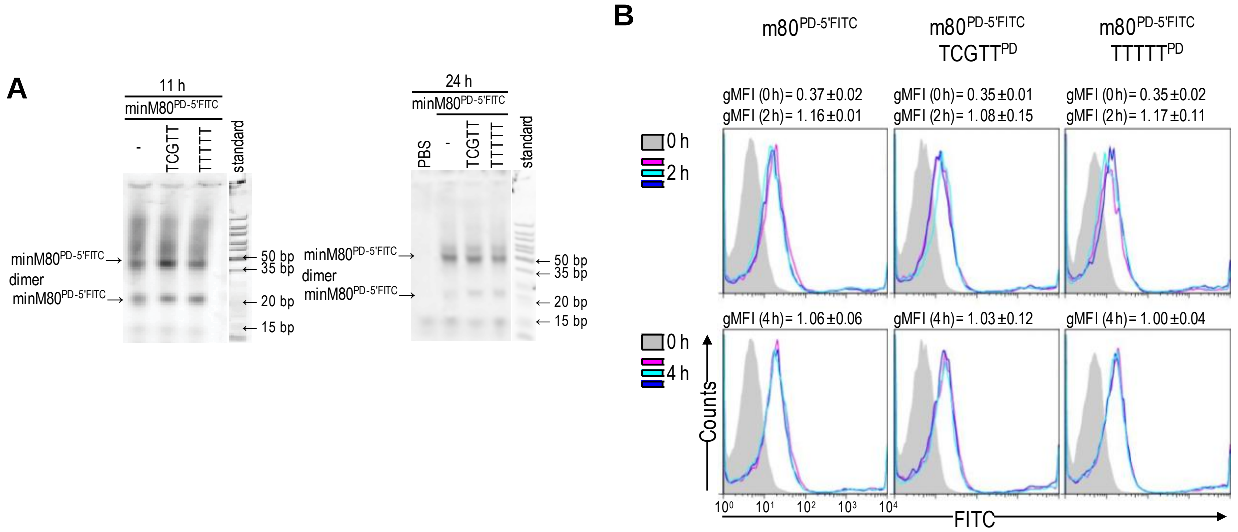

Fig 1.1*; part of* ***Supplemental Figure 9***. (**A**) The minM80$^{5'FITC}$ without and with TCGTT was added to cells and 11 and 24 h later the degradation of minM80$^{5'FITC}$ within cells was analyzed. (**B**) Short ODNs have no impact on internalization of ODN. Ramos Blue cells were treated with minM80$^{PD-5'FITC}$ without or with sODNs (TCGTT or TTTTT). At 0 h, and 2 and 4 h after treatment, the cells were collected, washed and the fluorescence intensity of minM80$^{PD-5'FITC}$ within the cell was measured using flow cytometry.

Next, a dose dependent analysis of the TLR9 activation for TCGTT at constant concentration of phosphodiester (PD) or phosphothioate (PTO)-based ODNs. The PTO-based ODNs and sODNs were used to validate the potential role of TCGTT on the degradation of ODN agonists by DNases since the PTO-

based oligonucleotides are resistant to degradation in comparison to PD-based agonists. Irrespective of the backbone of ODNs and sODNs, the TCGTT amplified TLR9 activation with both PD- and PTO-based ODNs (**Fig 1.2**). We think that this is the most convincing evidence against the role of sODNs in decreasing degradation of longer ODNs.

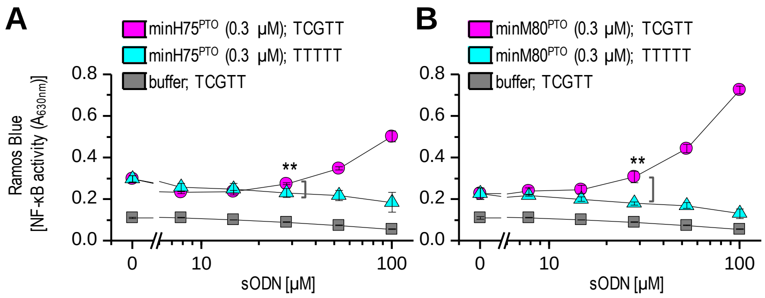

Fig 1.2*; part of* ***Supplemental Figure 8, Supplemental Figure 9***.
Dose response of TLR9 for sODN (PTO-backbone). Concentrations higher than 20 µM of TCGTT augment TLR9 activity by ODNs. Ramos Blue cells were stimulated with a combination of minH75^{PTO} (0.3 µM) (**A**) or minM80^{PTO} (0.3 µM) (**B**) and TCGTT^{PTO} (0-100 µM).

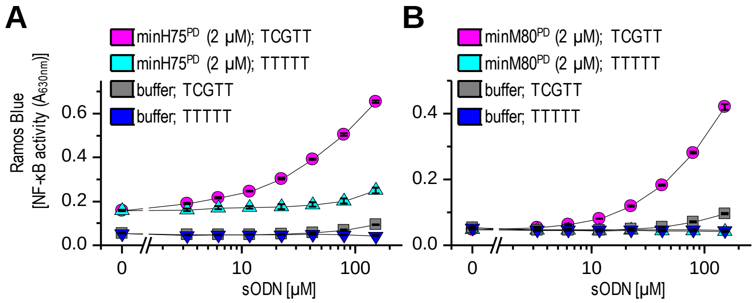

Fig 1.3*; part of* ***Figure 7*** *and* ***Supplemental Figure 8***.
Dose response of TLR9 for sODN (PD-backbone). Concentrations higher than 10 µM of TCGTT augment TLR9 activity by ODN. Ramos Blue cells were stimulated with a combination of minH75^{PD} (2 µM) (**A**) or minM80^{PD} (2 µM) (**B**) and TCGTT^{PD} (0-100 µM).

By dose dependent analysis we aimed to determine the lowest TCGTT concentration that augments the receptor activation by ODNs. In our results activation of B-lymphocytes was augmented already at concentrations higher than 10 µM TCGTT (**Fig 1.3**) for both minH75 and minM80. Moreover, amplification of TLR9 activation was proportional to the TCGTT concentration. For TTTTT, which could also serve as potential bait for nucleases, we observed no impact on the TLR9 activity augmentation.

The minimal required concentration of TCGTT that augments TLR9 activity by ODNs varies with respect to cell type and also with the type of ODN (**Supplemental Figure 2**). For BM-DCs (**Fig. 2**) the concentration of sODNs that already augments activation of TLR9 was 5 µM, which might not be surprising due to the physiological role of this cell type as the antigen presenting cells.

The concentration of sODNs required to augment TLR9 activity was considerably lower than the required concentration of the nucleotides reported to augment activity in the TLR8/ssRNA/uridine or TLR7/ssRNA/guanosine systems. For TLR8 and TLR7, the potentiation of activity was studied at 0.5 and 1 mM concentrations of uridine and guanosine, respectively. No dose response for guanosine/uridine has been reported for TLR7/TLR8 ; therefore, a direct comparison cannot be made. The reported *in vitro* binding affinities of guanosine to TLR7 and uridine to TLR8 are lower than concentrations used for the cell activation studies. The binding affinity of uridine for TLR8 is 55 µM(K_d) and the affinity of guanosine for TLR7 is 14 µM(K_d) that changes in the presence of ssRNA to approx. 1.0 µM; thus implying that nucleosides alone cannot activate TLR7 or TLR8 efficiently under physiological conditions, but the presence of the second binding site compensates for the relatively low affinity of nucleosides (Shibata et al., 2016; Tanji et al., 2015).

Taken together, those results support the conclusion that the CpG-containing short ssDNA fragments amplify activation efficiency of agonists by binding to TLR9 and not by effecting degradation and internalization.

Additional results were included in the manuscript and the discussion was amended. We also discuss in more detail the results in the light of recent findings on TLR7 and TLR8 (see point 1.4)

1.2) **Fig. 3B is not convincing or misunderstood by the reviewer**. The authors claim that DNase digestion products are enriched in the endosome. However, the gDNA input control that was loaded on the gel as control, already contains similar small fragments as the DNA isolated from endosomes. **What is the proof for fragment generation in the endosome?**

Firstly, the experiment was repeated with additional controls to enable better interpretation of results. Fragments shorter than 5-mers are free nucleotides (designated as FITC) that did not react in labeling with FITC-12-ddUTP (**Fig 1.4A**). With this experiment we demonstrated that the degradation products of dsDNA are localized in the endo/lysosomes and as such can serve as agonists for TLR9. Furthermore, we analyzed the degradation of FITC-labeled dsDNA in the cell lysate. With labeled DNA we could discriminate between host and exogenous DNA. dsDNA was labeled with FITC by PCR using FITC conjugated dUTP. Similar to the gDNA degradation, short fragments of the pre-labeled dsDNA were identified in the cell lysates of Ramos cells (**Fig 1.4B**). Moreover, the FITC-labeled dsDNA was degraded to some extend already in growth media (**Fig 1.4C**).

Taken together, short DNA fragments concentrate in the enriched endo/lysosomal fraction and as such could serve as agonists and ligands for TLR9. From results we could determine that degradation of dsDNA is a complex process that takes place outside the cells and proceeds within endo/lysosomes. The endosomal DNAse II digest dsDNA in endosomes and is required for TLR9 activation by dsDNA ligands (Chan et al., 2015). The text regarding degradation was formulated more precisely.

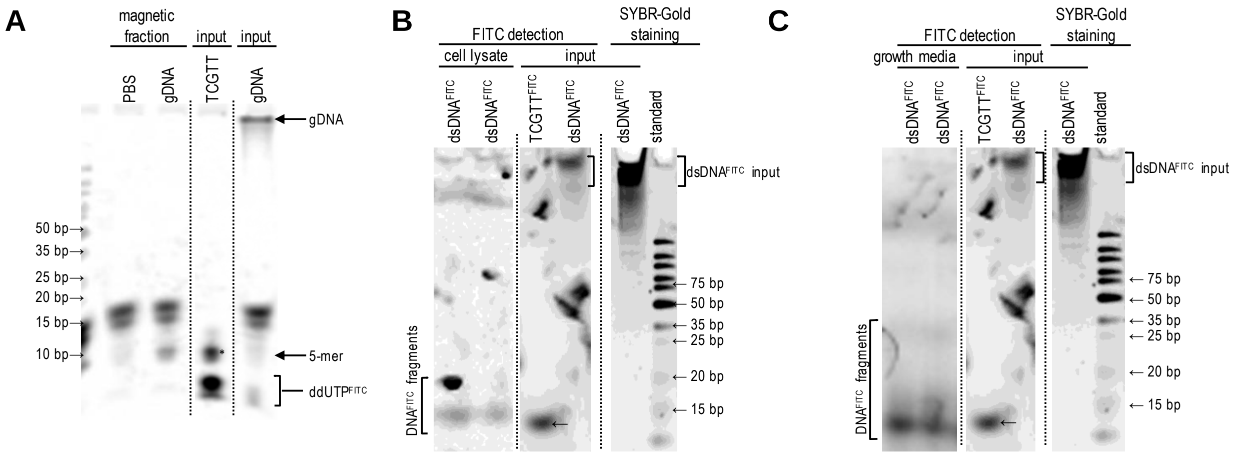

Figure 1.4*; part of* ***Figure 3***.
(**A**) Short DNA fragments originating from calf-thymus gDNA and endogenous human DNA were found in the enriched endo/lysosomal fractions as determined using a method combining the enrichment of endo/lysosomes by magnetic beads and labeling DNA with fluorophores. HEK293 cells were loaded with magnetic beads alone or together with calf thymus gDNA.
(**B**) Short DNA fragments from FITC-labeld dsDNA were detected in cell lysates. (**C**) Degradation of FITC-labeled dsDNA was detected already in growth media. Ramos cells were treated with FITC-labeled dsDNA and degradation products in growth media and in cell lysates were analyzed 24 h later.

1.3) **The binding studies of short ODNs with mouse TLR9-Fc does not show any binding specificity for the short ODN with the CG motif (Fig. 7A),** it also binds TTTTT ODNs. Shouldn't

according to the authors view, **the TCG bind better (or exclusively)**? The quality of the blot in Fig 7B is of poor quality and I was unable to judge it.

We perforemd the analysis of binding of ODN and sODN to TLR9-Fc (the immunoprecipitation). From given results, we can conclude that the binding of sODNs does not depend on agonist and pH (**Figure** 7A). We found that the amount of TCGTT bound to TLR9/ODN was higher than the amount of TTTTT (*three independent experiments*); however, we could not deduce whether the binding affinity of TTTTT and TCGTT differs even though the augmentation of TLR9 relies on the presence of CG dinucleotide of the sODN (**Fig 1.5**).

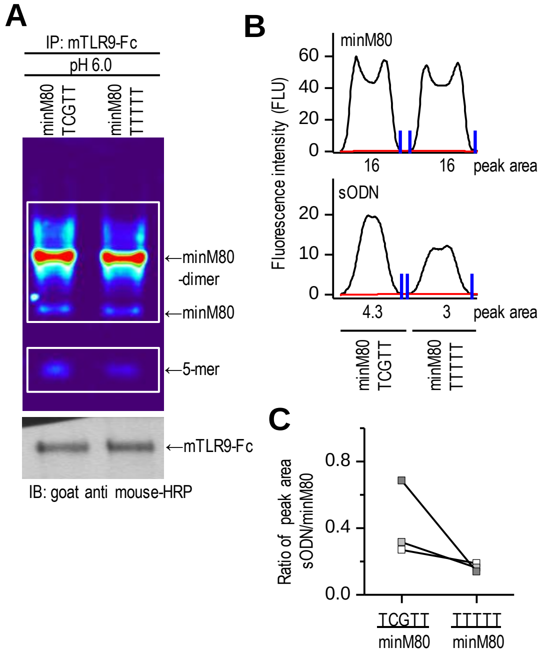

Fig 1.5; *part of* ***Supplemental Figure 9***.
At equal loading of TLR9-Fc to Protein-G conjugated beads the amount of bound TCGTT (based on the fluorescence intensity of eluted sODN) was higher than that of TTTTT (**C**) and was independent of the presence of ODN and pH (*see also* ***Fig. 7***).
ODN minM80^{FITC} (250 pmol) and/or sODNs (TCGTT^{FITC} or TTTTT^{FITC} (5 µmol)) were added to the Protein-G-conjugated beads with bound mTLR9-Fc or control beads. The DNA fragments are visualized as an intensity heat map (**A**). Fluorescence intensity profile (**B**) within marked area (**A**) is given and peak area calculated (**B**). mTLR9-Fc bound to Protein-G beads was determined by Western blot analysis using secondary goat anti mouse-HRP antibody detection.

1.4) In the **discussion the authors could elaborate more on the similarities (and differences) on TLR8 and TLR9** structure in regard to binding pockets for small CG containing fragments.

Discussion related to recent findings on the structure of TLR7 and TLR8 with natural ligand and degradation product was expanded as proposed by the reviewer. (Marked yellow in the Discussion)

Reviewer #2 (Remarks to the Author):

In the manuscript entitled ‘Short single-stranded DNA degradation products augment the activation of Toll-like receptor 9’ Pohar and colleagues report the observation, that short CG containing DNA can increase the response of TLR9 towards longer oligonucleotides.

This observation is novel and worth reporting and overall topic is of interest to the readership of Nature Communications. Overall I am supportive of publishing this article.

1) I have a conceptual issue that needs to be addressed. The TLR9/ODN co-crystal structure with immune stimulatory ODNs shows a 12 nucleotide ODN binding to TLR9 in a 2:2 stoichiometry. Importantly, the structure demonstrated that the integrity of the ODN is important since one binding side on one TLR9 recognizes the CpG motif, while another binding site on the dimerization partner recognizes the ODN backbone**. I think what the authors wish to claim regarding the putative mechanism how the short ODNs function is misleading.** They speculate that short ODNs bind to a TLR9 dimer that is already crosslinked by a single stimulatory ODN and that thereby the short ODNs would enhance TLR9 activation only if sub-optimal ligand concentration of the longer ODN is present.

If such a model is correct, then **larger amounts of the sODNs should even inhibit the responses to long ODN**, because the ODNs compete for the same binding site. This mode would be different from the cooperative activation mode of TLR8 where two binding sites are postulated, one of which acts allosterically.

The authors should address **this point experimentally to work out the mechanisms in more detail**. Otherwise the paper stays very descriptive.

We agree with the reviewer that the sODN might be able to bind to the same site as the agonistic 12-mer ODN; however at the substantially lower affinity. If it were to bind to the same site as the ODN it might be able to inhibit activation, although perhaps at very high concentration. However the existence of an additional binding site for sODNs with allosteric effect on TLR9 can explain the results we obtained.

While it is difficult to conclusively identify the site of action of the sODNs in the absence of a crystal structure, the effect of sODN on TLR9 activation strongly resembles the effect of mono- and dinucleotides on TLR7 and TLR8. Since the augmentation of activity is strongest at lower concentrations of the ODN the sODNs might also stabilize the complex at suboptimal 2:1 (TLR9:ODN) ratio, when only a single interactions interface TLR9:ODN:TLR9 is formed.

In order to substantiate the proposed model of TLR9 activation by ODN and TCGTT we performed a detailed dose response of TLR9 activation analysis for ODNs and sODNs (**Fig 2.1**). Results of the dose response analysis support the hypothesis that the binding site for TCGTT might differ from the binding site for CpG dinucleotide of agonist (**Fig 2.1A,B**). We showed that increasing the concentration of TCGTT, which by itself fails to activate TLR9, amplifies activation of the receptor by the CpG-containing oligonucleotides (importantly, also non-stimulatory ODNs). Furthermore, the TLR9 dose response curves titrating ODNs at constant concentration of TCGTT support the notion that TCGTT has an allosteric effect on TLR9 activation with the agonistic ODN and acts synergistically in combination with inactive CpG-containing longer ssDNA fragments. Indeed the fold of activation has a maximum at 1-2 μM, depending on the ODN.

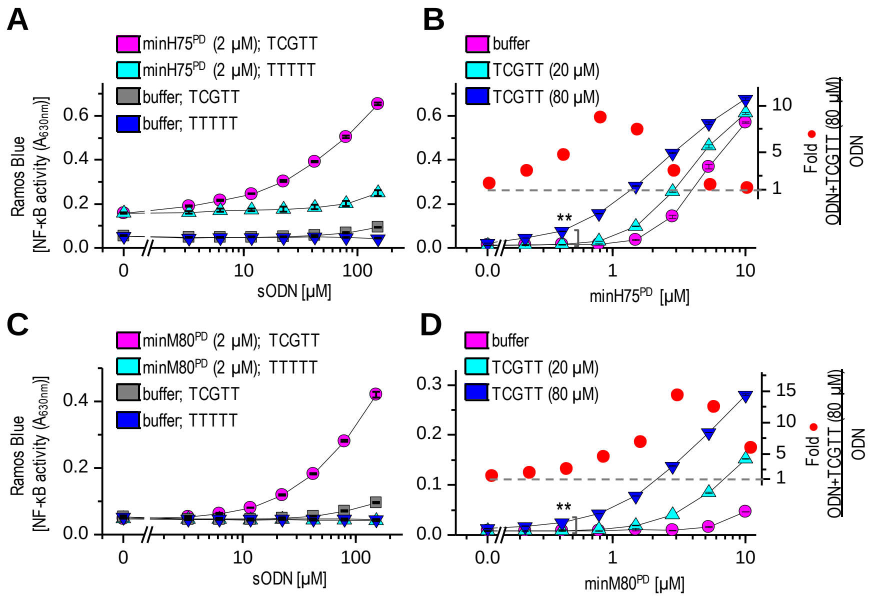

Fig 2.1*; part of* ***Figure 7*** *and* ***Supplemental Figure 8****.*
(**A,C**) Dose response of hTLR9 activation for sODN^{PD} (0-160 µM) at constant concentration of ODN^{PD} (minH75^{PD} or minM80^{PD}; 2 µM).
(**B,D**) Dose response of hTLR9 activation for ODN^{PD} (minH75^{PD} or minM80^{PD}; 0-10 µM) at constant concentrations of TCGTT^{PD} (20, 80 µM). NF-κB/AP-1-dependent SEAP expression was analyzed 18 h later.

Based on our results we propose a similar mechanism of activation for TLR9 as for the TLR7 and TLR8 (*described in the Discussion*). The interaction surface of TLR9 for TCGTT is smaller than for the 12-mer ODN, similar as for the uridine/guanosine and TLR8/TLR7. The TCGTT itself cannot induce formation of the activated TLR9 form even though it binds to the receptor (**Figure 7, Supplemental Figure 8**), whereas the synergistic cooperation of TCGTT and longer oligonucleotide augments the activity. Although ODN-binding to the primary site directly induces the activated form of the TLR9 dimer, binding of TCGTT can allosterically promote TLR9 activation, stabilizing TLR9-ODN dimer. The augmentation is proportional to the concentration of sODNs, suggesting that TCGTT binds to TLR9 at different position as the CpG motif of agonists. Moreover, a dose response of TLR9 at constant concentration of TCGTT and increasing concentration of agonists suggests allosteric activation of TLR9 by TCGTT for ODNs.

The stabilization of TLR9-ODN is especially important for TLR9 activation with ODNs with e.g. methylated CpG motifs (**Figure 6A**) and with CpG-containing ODNs which fail to activate TLR9 (e.g. combination of human TLR9 and mouse-specific ODN) (**Fig 2.1C,D**, **Figure 5**).

Taken together, we propose an extended model of TLR9 activation which considers the role of short CG-containing oligonucleotides (**Fig 2.2**). The sODNs bind to TLR9 independent of the ODN agonists and strengthen the active ODN-TLR9 complex. The effect of sODNs on TLR9 activity was detected for the mammalian gDNA and bacterial DNA (*described below-next point*). A physiological relevance of sODNs is nonnegligible, especially in combination with the elevated concentrations of cell free host methylated DNA.

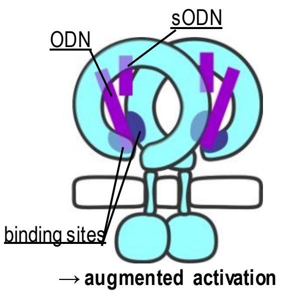

Figure 2.2*; part of* ***Figure 7****.*
The proposed extended model of binding sODN and ODN to TLR9.

Description of the results and the proposed model, and the discussion were included in the text.

2) Another suggestion to make the observation reported in this paper more relevant is **to study the ODN sizes appearing upon viral infection** and/or to study whether **ODNs would augment the responses towards natural TLR9 stimuli**.

In order to elucidate an effect of sODNs on activation of TLR9 with the natural TLR9 stimuli, we selected three bacteria genomic DNA *Escherichia coli* and *Pseudomonas aeruginosa* that differ in the contents of CpG motifs: 7.3 and 12 %, respectively (Dalpke et al., 2006) (Pohar et al., 2017). The TCGTT augmented activation of TLR9 by isolated bacterial DNA (**Fig 2.3**). Experiments with the use of whole bacteria are not relevant because of the presence of other PAMPs (LPS, flagellin) that can activate additional signaling pathways.

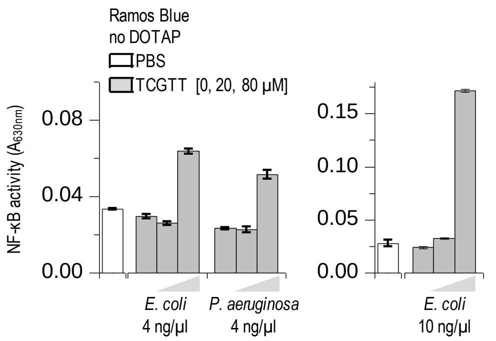

Fig 2.3; *part of* ***Figure 6***.
TLR9 activation with DNA isolated from bacteria is augmented by TCGTT. Ramos Blue cells were stimulated with DNA from bacteria *P. aeruginosa* (4 ng/µl) and *E. coli* (4 and 10 ng/µl) and TCGTT.
NF-κB/AP-1-dependent SEAP expression was analyzed 18 h later.

In addition to the bacterial DNA, we examined the effect of TCGTT on the TLR9 activation by mammalian gDNA isolated from mouse fibroblasts NIH3T3 and by unmethylated gDNA. Random PCR amplification of gDNA was used to obtain unmethylated mammalian gDNA (Burge et al., 1992; Jabbari and Bernardi, 2004; Stacey et al., 2003; Uccellini et al., 2008). The sODNs also augmented activation of TLR9 by mammalian gDNA and unmethylated dsDNA, (**Fig 2.4**) indicating that the augmentation of TLR9 activation by short CG-containing ssDNA fragments is important also for the natural ligands, e.g. CpG-containing DNA.

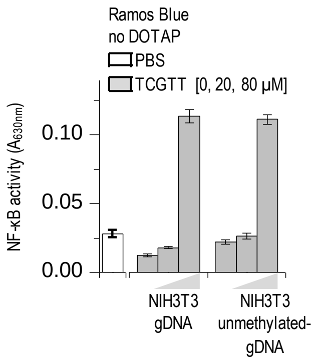

Fig 2.4; *part of* ***Figure 6***.
sODN TCGTT augments the activation of hTLR9 by mammalian gDNA. Ramos Blue cells were stimulated with gDNA isolated from the mouse fibroblasts NIH3T3, or with PCR-amplified gDNA from the NIH3T3 cells (unmethylated gDNA) (10 ng/µl).
NF-κB/AP-1-dependent SEAP expression was analyzed 18 h later.

Burge, C., Campbell, A.M., and Karlin, S. (1992). Over- and under-representation of short oligonucleotides in DNA sequences. Proc. Natl. Acad. Sci. U. S. A. *89*, 1358–1362.

Chan, M.P., Onji, M., Fukui, R., Kawane, K., Shibata, T., Saitoh, S., Ohto, U., Shimizu, T., Barber, G.N., and Miyake, K. (2015). DNase II-dependent DNA digestion is required for DNA sensing by TLR9. Nat. Commun. *6*, 5853.

Dalpke, A., Frank, J., Peter, M., and Heeg, K. (2006). Activation of toll-like receptor 9 by DNA from different bacterial species. Infect. Immun. *74*, 940–946.

Jabbari, K., and Bernardi, G. (2004). Cytosine methylation and CpG, TpG (CpA) and TpA frequencies. Gene *333*, 143–149.

Pohar, J., Yamamoto, C., Fukui, R., Cajnko, M.M., Miyake, K., Jerala, R., and Benčina, M. (2017). Selectivity of human TLR9 for double CpG motifs and implications for the recognition of genomic DNA. J. Immunol.

Shibata, T., Ohto, U., Nomura, S., Kibata, K., Motoi, Y., Zhang, Y., Murakami, Y., Fukui, R., Ishimoto, T., Sano, S., et al. (2016). Guanosine and its modified derivatives are endogenous ligands for TLR7. Int. Immunol. *28*, 211–222.

Stacey, K.J., Young, G.R., Clark, F., Sester, D.P., Roberts, T.L., Naik, S., Sweet, M.J., and Hume, D.A. (2003). The Molecular Basis for the Lack of Immunostimulatory Activity of Vertebrate DNA. J. Immunol. *170*, 3614–3620.

Tanji, H., Ohto, U., Shibata, T., Taoka, M., Yamauchi, Y., Isobe, T., Miyake, K., and Shimizu, T. (2015). Toll-like receptor 8 senses degradation products of single-stranded RNA. Nat. Struct. Mol. Biol. *22*, 109–115.

Uccellini, M.B., Busconi, L., Green, N.M., Busto, P., Christensen, S.R., Shlomchik, M.J., Marshak-Rothstein, A., and Viglianti, G.A. (2008). Autoreactive B Cells Discriminate CpG-Rich and CpG-Poor DNA and This Response Is Modulated by IFN-. J. Immunol. *181*, 5875–5884.
