# Supplementary material for: Short single-stranded DNA degradation products augment the activation of Toll-like receptor 9
Source: Nat Commun. 2017 May 22;8:15363. doi: 10.1038/ncomms15363 (PMC5458134; doi:10.1038/ncomms15363)
Supplement: Peer Review File [file ncomms15363-s2.pdf]

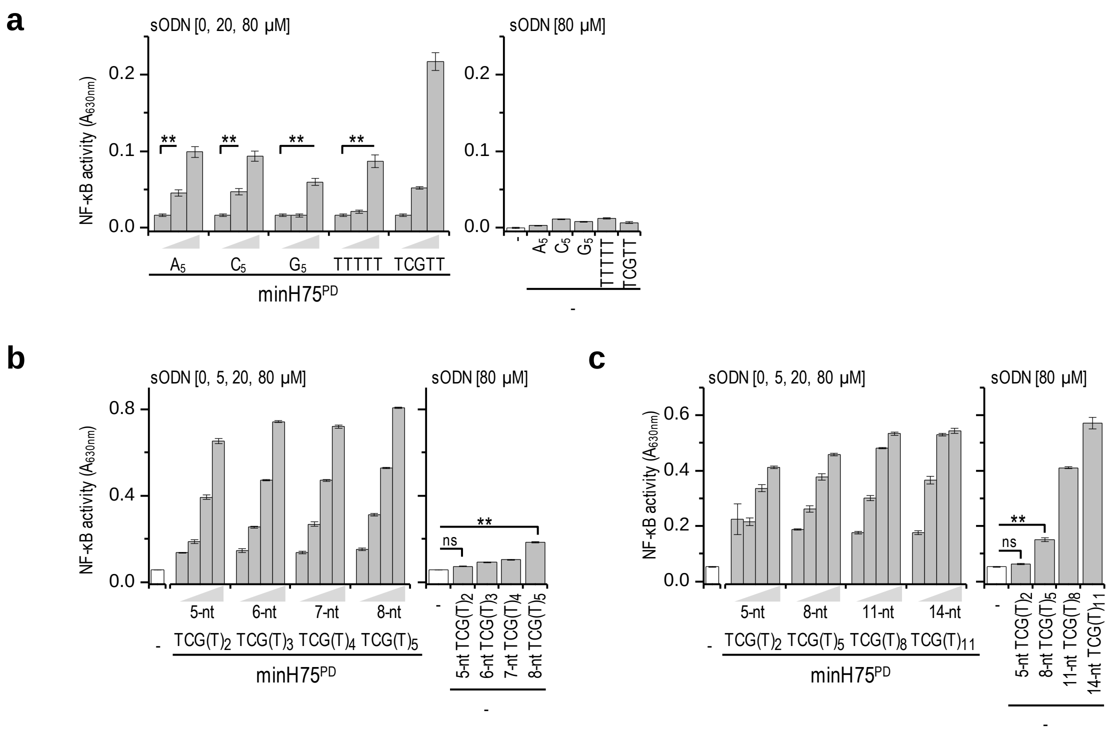

Supplementary Figure 1, related to **Fig. 1. sODNs longer than 7 nt activate hTLR9 at high concentrations.**

(**a**) The sODNs[PD] TTTTT, A_5, C_5, and G_5 only slightly improve TLR9 activation with minH75[PD].

(**b**) 6 nt and 7 nt long sODNs enhance the activation of hTLR9 when co-administered with minH75 and have a minor effect when administered alone.

(**c**) At high concentrations, sODNs longer than 7 nt activate hTLR9 and, as such, contribute to the enhanced activation of TLR9. Ramos Blue cells were stimulated with a mixture of minH75[PD] and sODNs[PD] (0, 5, 20, 80 µM) or with only sODNs[PD] (80 µM) as a control.

(**a-c**) The NF-κB/AP-1-regulated expression of SEAP was analyzed 18 h after stimulation. (Data are representative of three independent experiments. Bars represent the means of five biological replicates ±s.d.; **$p<0.05$, ns $p>0.05$, unpaired two-tailed Student t-test).

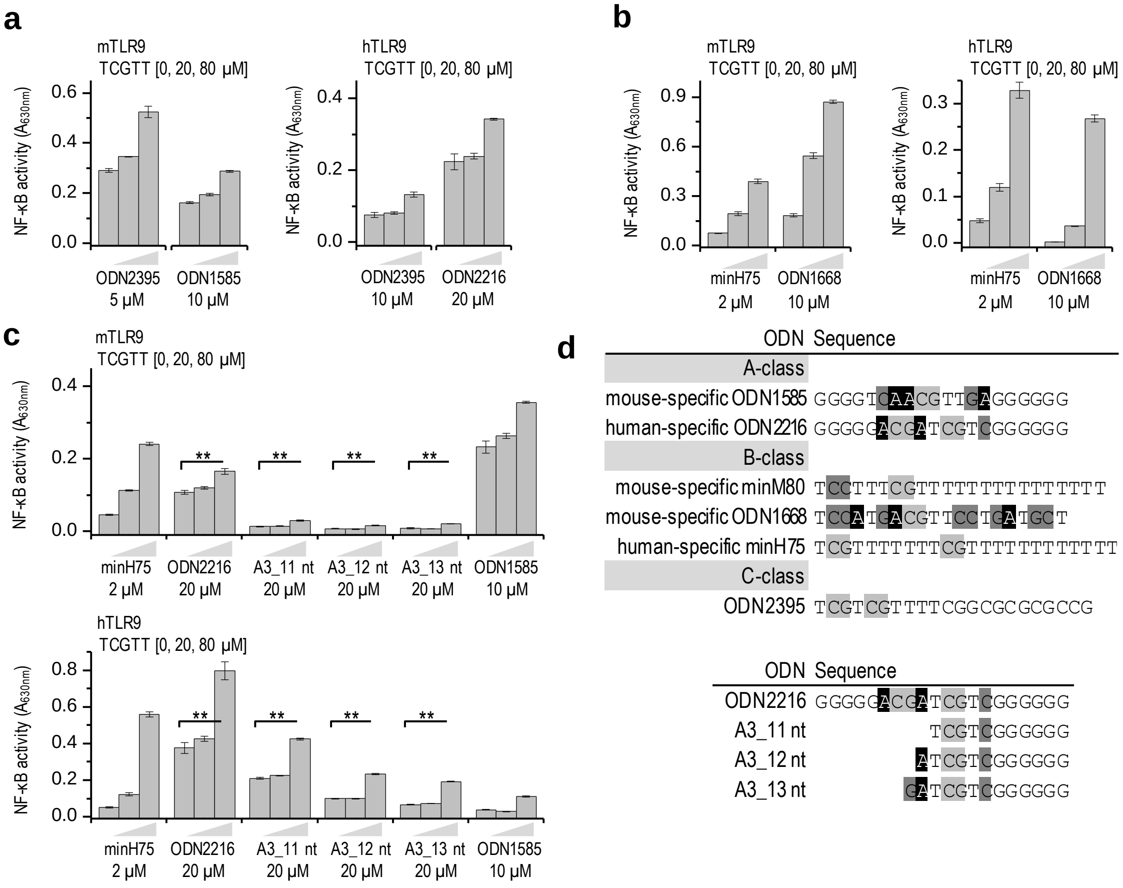

Supplementary Figure 2, related to **Fig. 2. TCGTT improves the activation of TLR9 with A-, B-, and C-class ODNs.**

TCGTT improves mTLR9 and hTLR9 activation with (**a**) the A- and C-class ODNs, (**b**) ODN1668, and (**c**) shorter variants of ODN2216. HEK293-Blue cells expressing mTLR9 or hTLR9 were stimulated with C-class ODN2395^{PD}, A-class mouse-specific ODN1585^{PD}, human-specific ODN2216^{PD}, B-class minH75^{PD}, minM80^{PD}, mouse-specific ODN1668^{PD}, shorter ODN2216^{PD} variants, and TCGTT^{PD} (0, 20, 80 µM). NF-κB/AP-1-regulated SEAP expression was analyzed 18 h after stimulation; shown are the activities after subtracting the intrinsic activity of non-stimulated cells. (Data are representative of two independent experiments. Bars represent the means of four biological replicates ±s.d.; **p<0.05, unpaired two-tailed Student t-test)

(**d**) Sequences of ODNs.

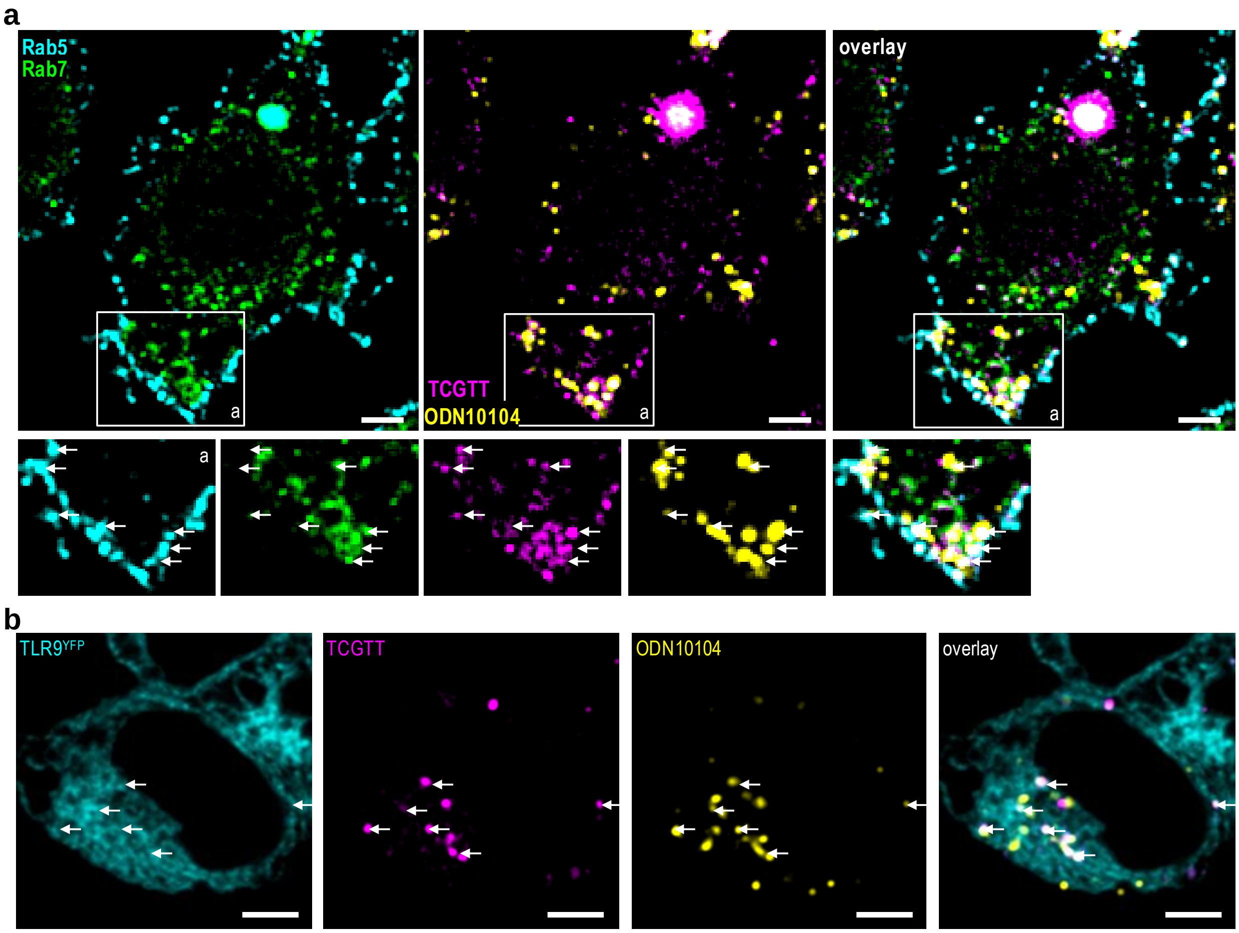

Supplementary Figure 3, related to **Fig. 3. Colocalization of ODN and sODN with hTLR9.**

HEK293 cells were transfected with (**a**) hTLR9 or (**b**) hTLR9^{YFP} and treated with 5 µM TCGTT$^{PD\text{-}3'Cy3}$ and 3 µM human-specific ODN10104$^{PD\text{-}3'Cy5}$. Eighteen h later, the spatial distribution of ODN and sODN was analyzed (**a**) in cells stained for Rab5 and Rab7 with anti-Rab5^{ATTO390} and anti-Rab7^{AlexaFluor488} antibodies and in cells transfected with (**b**) the chimera hTLR9^{YFP}. Arrows mark regions of ODN10104 and TCGTT colocalization with (**a**) Rab5 or Rab7 or (**b**) hTLR9^{YFP}. (Data are representative of three independent experiments.). Scale bar = 5 µm.

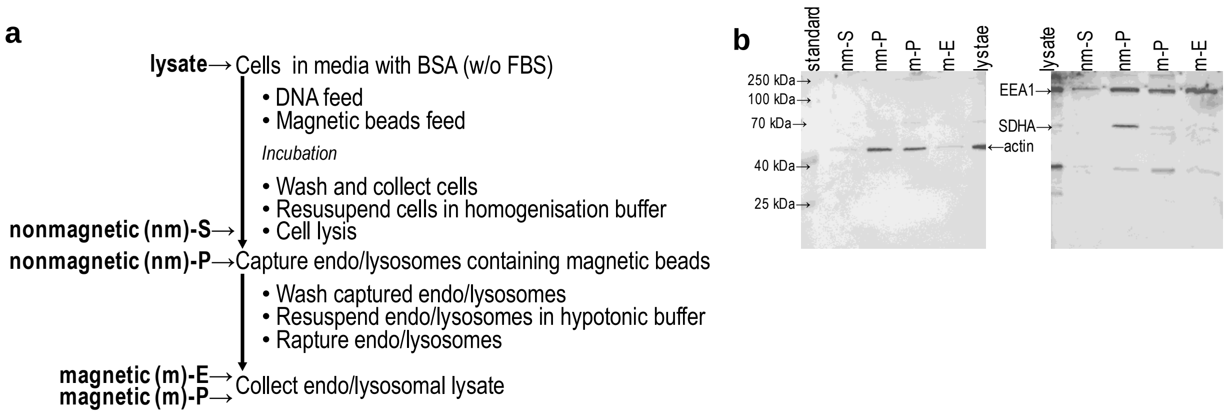

Supplementary Figure 4, related to **Fig. 3. Enrichment of endo/lysosomal fraction.**

(**a**) Flowchart of endo/lysosomal fraction-enrichment steps with magnetic particles.

(**b**) Western blots at various purification steps, stained for EEA1 (endosomal early antigen 1), SDHA (succinate dehydrogenase complex, subunit A), and actin. Legend: nm-P and nmS, non-magnetic pellet and supernatant after removing magnetic endo/lysosomal fraction; m-E and m-P, magnetic supernatant and pellet of enriched endo/lysosomal fraction.

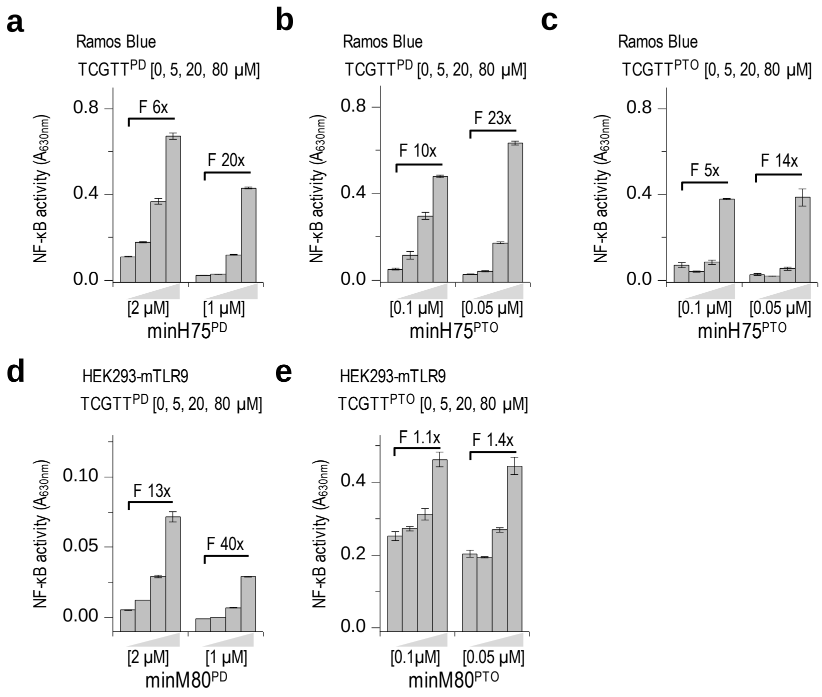

Supplementary Figure 5, related to **Fig. 4. Augmentation of TLR9 response by sODN is most potent for weak ODN agonists.**

Ramos Blue cells were stimulated with (**a**) minH75^{PD} and TCGTT^{PD}, (**b**) minH75^{PTO} and TCGTT^{PD}, or (**c**) minH75^{PTO} and TCGTT^{PTO} (minH75^{PTO} (0.1 μM), shown as **Fig. 2b,** is used for these calculations). NF-κB/AP-1-regulated SEAP expression was measured 18 h after stimulation; the activities after the subtraction of the intrinsic activity of non-stimulated cells are shown. Note: the augmentation fold (F) of TLR9 response with sODN is the highest at subthreshold concentrations of minH75.

HEK293-Blue cells expressing mTLR9 were stimulated with (**d**) minM80^{PD} and TCGTT^{PD} or (**e**) minM80^{PTO} and TCGTT^{PTO}. The fold change (F) was calculated as the ratio between the highest TLR9 activity and the TLR9 activity when only ODN was added. The activities after the subtraction of the intrinsic activity of non-stimulated cells are shown. (Data are representative of two independent experiments. Bars represent the means of five biological replicates ±s.d.).

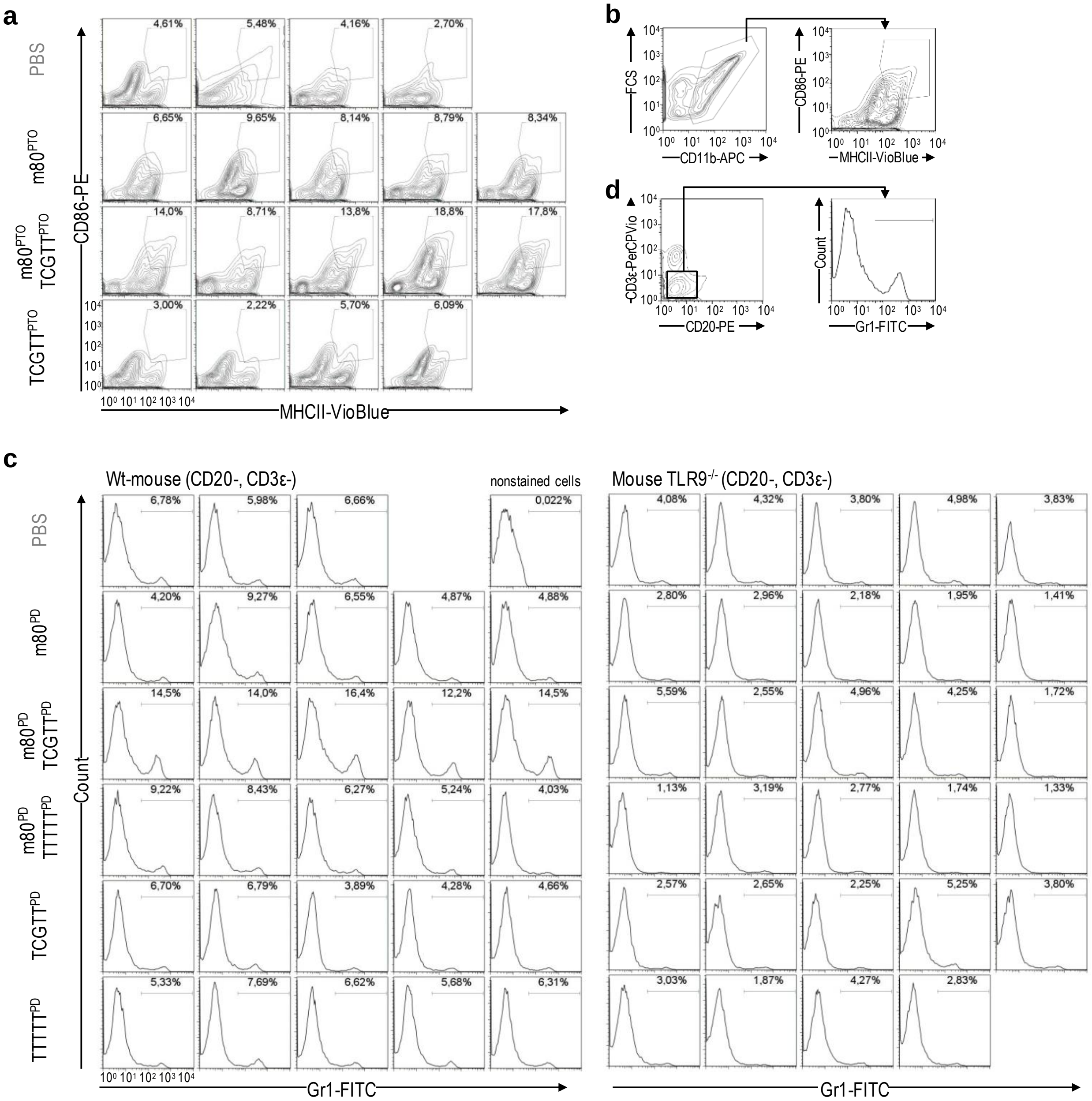

Supplementary Figure 6, related to **Fig. 5.**

(**a**, **b**) **The combination of sODN (TCGTT) and TLR9-agonistic ODN (minM80) induces the expression of MHCII and CD86.** Mice (wt) were treated with PBS or minM80^{PTO} (40 nmol) with and without TCGTT^{PTO} (1 µmol). Twenty-four h later, the peritoneal lavage was collected, and the cells were stained for cell surface markers MHCII (MHCII-VioBlue) and CD86 (CD86-PE). The percentages of cells with expressed CD86^{high} and MHCII^{high} were analyzed. (**b**) Gating strategy. Cells positive for CD11b were analyzed for expression of CD86 and MHCII. (**c**, **d**) The mixture of TCGTT^{PD} and minM80^{PD} induces TLR9-dependent granulocyte infiltration into the spleen. Mice (wt and TLR9$^{-/-}$) were treated with PBS and minM80^{PD} (120 nmol) with or without TCGTT^{PD} (1.2 µmol) or TTTTT^{PD} (1.2 µmol). Twenty-four h later, the mice were sacrificed; the spleenocytes were stained for cell surface markers Gr1 (GR1-FITC), CD20 (CD20-PE), and CD3ε (CD3ε-PerCpVio700). Cells negative for CD20 and CD3ε were analyzed for Gr1-positivity. (**d**) Gating strategy for (**c**).

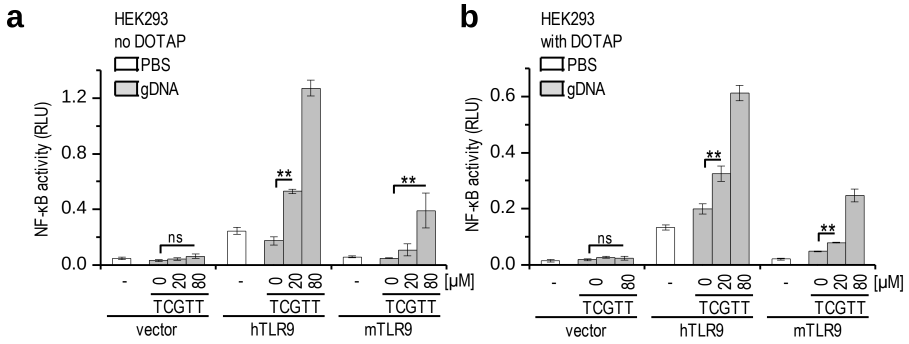

Supplementary Figure 7. sODN enhances the activation of hTLR9 and mTLR9 by genomic DNA.

TCGTT augments the activation of HEK293 cells transfected with hTLR9 or mTLR9 stimulated with gDNA. HEK293 cells were stimulated with calf thymus gDNA (8 ng/µl) with DOTAP (DNA:DOTAP ratio 1:3) (**a**) or calf thymus gDNA (15 ng/µl) without DOTAP (**b**) in combination with TCGTT^{PD}. NF-κB-dependent luciferase activity was measured 18 h after stimulation. (Data are representative of two independent experiments. Bars represent the means of five biological replicates ±s.d.; **$p<0.05$, ns $p>0.05$, unpaired two-tailed Student t-test).

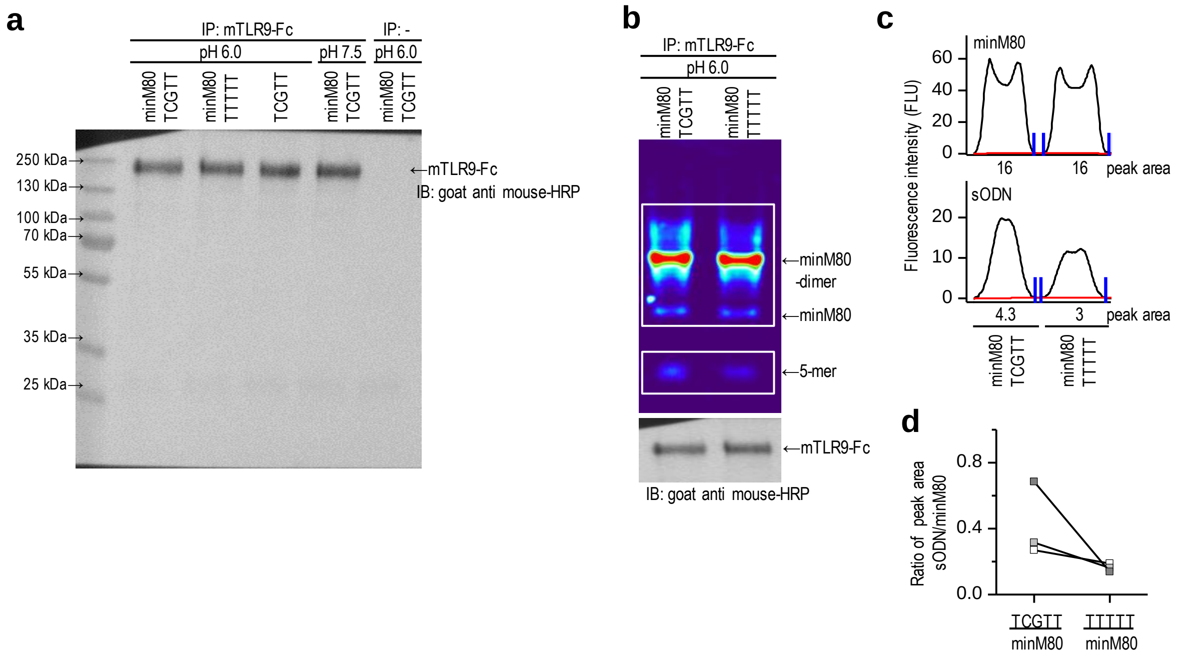

Supplementary Figure 8; related to **Fig. 7. TLR9-Fc binds TCGTT^{FITC} better than TTTTT^{FITC} at constant concentrations of minM80^{FITC}.**

(**a**) mTLR9-Fc bound to Protein-G-beads was determined with Western blot analysis using secondary goat anti mouse-HRP antibody detection.

(**b**, **c**) Fluorescence intensity profile (**c**) within marked area of separated ODNs and sODN (**b**) was established and the peak area was calculated. (**d**) Ratio of peak areas of sODN and minM of TCGTT *vs.* TTTTT is presented. (Results of three independent biological experiments were performed.).

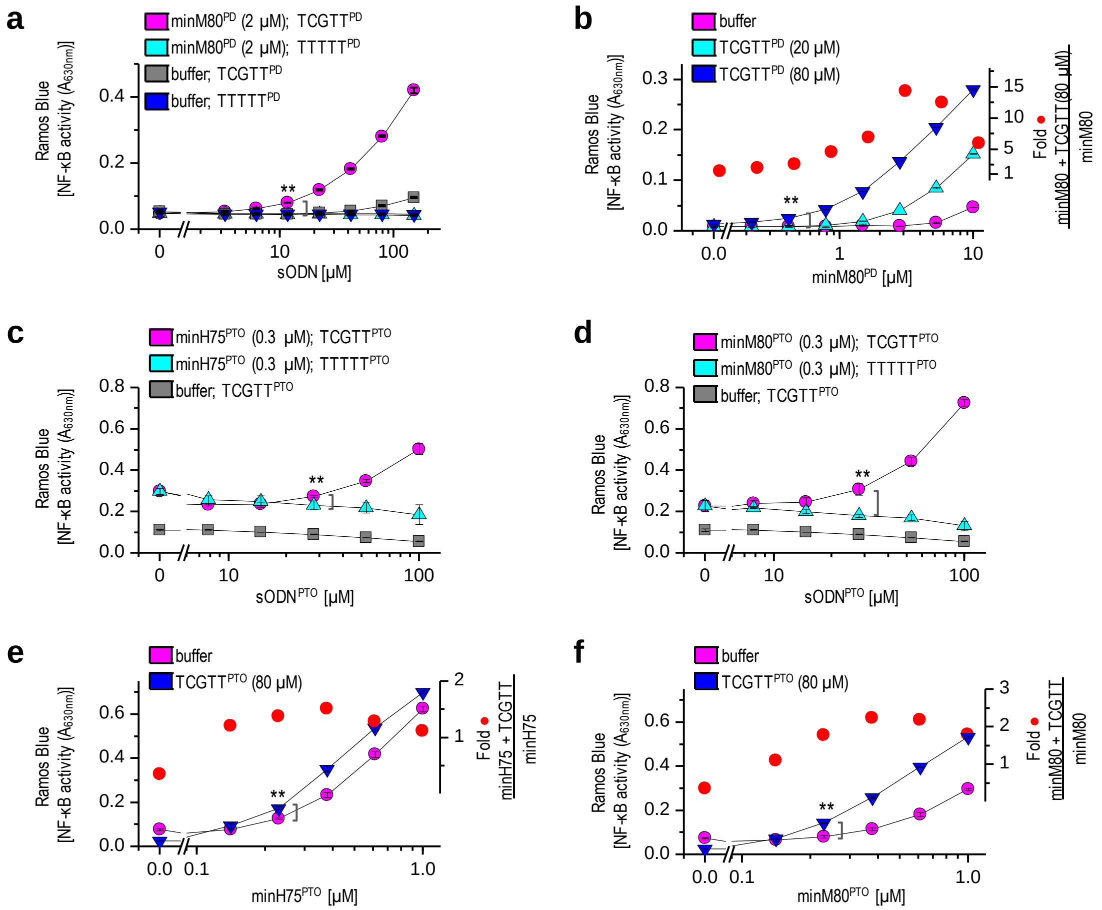

Supplementary Figure 9. The sODNs binds to TLR9.

(**a**,**b**) Dose response of hTLR9 for (A) sODNs (0-160 µM, TCGTT or TTTTT) at constant concentration of minM80^{PD} (2 µM) and (B) for minM80^{PD} (0-10 µM) at constant concentrations of TCGTT (20, 80 µM).

(**c**-**f**) Dose response of hTLR9 for (**c**,**d**) PTO-based sODNs (0-100 µM; TCGTT^{PTO} or TTTTT^{PTO}) at constant concentration of ODN^{PTO} (2 µM; minH75^{PTO} or minM80^{PTO}) and (**e**,**f**) for ODN^{PTO} (0-1 µM; minH75^{PTO} or minM80^{PTO}) at constant concentrations of TCGTT (80 µM).

(**a**-**f**) Ramos Blue cells were stimulated with mixtures of ODNs^{PTO} and sODNs^{PTO}. NF-κB/AP-1-dependent SEAP expression was analyzed 18 h later. Fold change in the TLR9 activity was calculated as ratio between SEAP expressions triggered by ODN with TCGTT (80 µM) and ODN. (Data are representative of two independent experiments. The means of three biological replicates ±s.d. are shown; **p<0.05, unpaired two-tailed Student t-test).

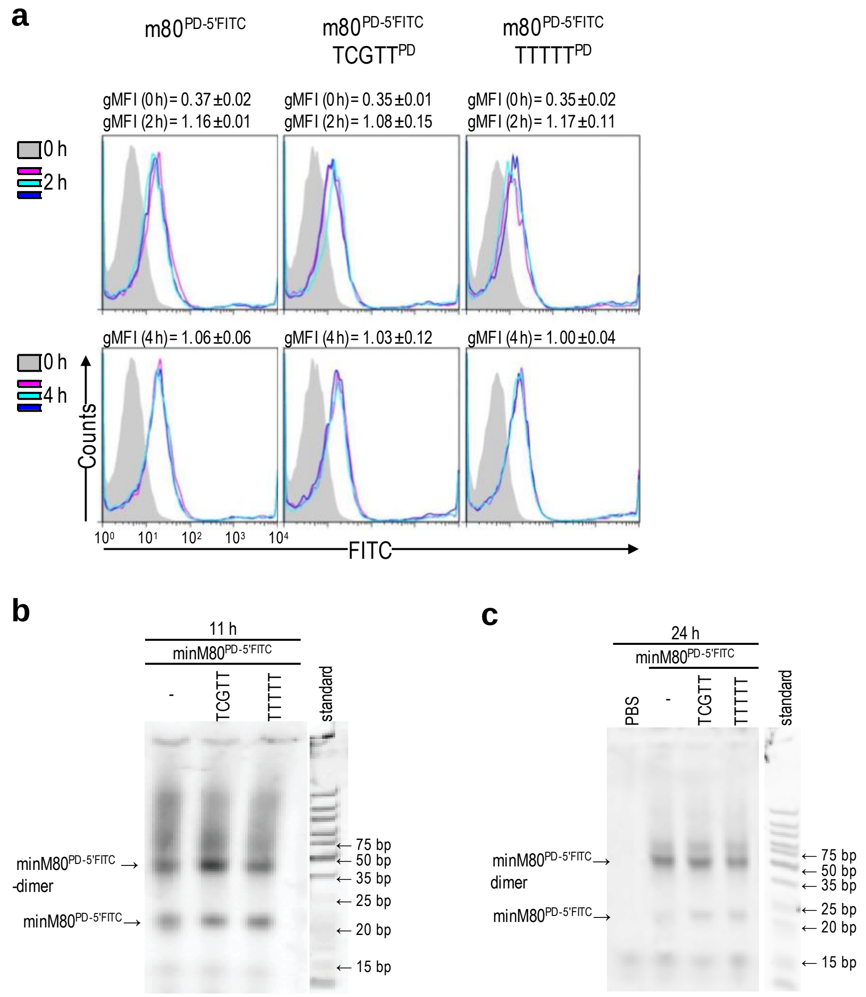

Supplementary Figure 10. The sODNs had no impact on internalization of ODN and degradation.

(**a**) Ramos Blue cells treated with minM80$^{PD\text{-}5'FITC}$ without or with sODNs (TCGTT^{PD}, TTTTT^{PD}). The internalization of FITC-labeled ODN at 0, 2 h and 4 h was determined with flow cytometry. (Results of all biological replicas are shown. Geometric mean of fluorescence intensity (gMFI) is calculated from three biological replicates ±s.d.).

(**b,c**) Intracellular degradation of ODN is not effected by sODN. Ramos Blue cells (cultured in IMDM with or without FBS were treated with minM80$^{PD\text{-}5'FITC}$ (5 µM) without or with sODNs (TCGTT^{PD}, TTTTT^{PD}) (80 µM). The cells were collected 11 h (**b**) and 24 h (**c**) later, lysed and content of FITC-labeled oligonucleotides was analyzed.
